# Comparative case study on NAMs: towards enhancing specific target organ toxicity analysis

**DOI:** 10.1007/s00204-024-03839-7

**Published:** 2024-08-29

**Authors:** Kristina Jochum, Andrea Miccoli, Cornelia Sommersdorf, Oliver Poetz, Albert Braeuning, Tewes Tralau, Philip Marx-Stoelting

**Affiliations:** 1 Department of Pesticides Safety, German Federal Institute for Risk Assessment, Berlin, Germany; 2 Institute for Marine Biological Resources and Biotechnology (IRBIM), National Research Council, Ancona, Italy; 3 Signatope GmbH, Tübingen, Germany; 4 NMI Natural and Medical Sciences Institute at the University of Tübingen, Reutlingen, Germany; 5 Department of Food Safety, German Federal Institute for Risk Assessment, Berlin, Germany

**Keywords:** HepaRG, RPTEC, Omics, Pathway analysis, Gene enrichment, Pesticides

## Abstract

Traditional risk assessment methodologies in toxicology have relied upon animal testing, despite concerns regarding interspecies consistency, reproducibility, costs, and ethics. New Approach Methodologies (NAMs), including cell culture and multi-level omics analyses, hold promise by providing mechanistic information rather than assessing organ pathology. However, NAMs face limitations, like lacking a whole organism and restricted toxicokinetic interactions. This is an inherent challenge when it comes to the use of omics data from in vitro studies for the prediction of organ toxicity in vivo. One solution in this context are comparative in vitro–in vivo studies as they allow for a more detailed assessment of the transferability of the respective NAM data. Hence, hepatotoxic and nephrotoxic pesticide active substances were tested in human cell lines and the results subsequently related to the biology underlying established effects in vivo. To this end, substances were tested in HepaRG and RPTEC/tERT1 cells at non-cytotoxic concentrations and analyzed for effects on the transcriptome and parts of the proteome using quantitative real-time PCR arrays and multiplexed microsphere-based sandwich immunoassays, respectively. Transcriptomics data were analyzed using three bioinformatics tools. Where possible, in vitro endpoints were connected to in vivo observations. Targeted protein analysis revealed various affected pathways, with generally fewer effects present in RPTEC/tERT1. The strongest transcriptional impact was observed for Chlorotoluron in HepaRG cells (increased *CYP1A1* and *CYP1A2* expression). A comprehensive comparison of early cellular responses with data from in vivo studies revealed that transcriptomics outperformed targeted protein analysis, correctly predicting up to 50% of in vivo effects.

**Supplementary Information:**

The online version contains supplementary material available at 10.1007/s00204-024-03839-7.

## Introduction

Given the at times heated discussions about regulatory toxicology in the political and public domain, the quite remarkable track record of toxicological health protection sometimes tends to go unnoticed. Not only are chemical scares such as the chemically induced massive acute health impacts in the 1950ies, 60ies and 70ies a thing of the past (Herzler et al. 2021), but in many parts of the world, there are now regulatory frameworks in place which aim at the early identification of potential health risks from chemicals. Within Europe, the most notable in terms of impact are probably REACH (EC 2006) and the regulations on pesticides (EC 2009) both of which still overwhelmingly rely on animal data for their risk assessments. This has manifold reasons, one being the historical reliability of animal-based systems for the prediction of adversity in humans. However, there are a number of challenges to this traditional approach. These comprise capacity issues when it comes to the testing of thousands of new or hitherto untested substances, the testing of mixtures, the ever-daunting question of species specificity or the limitation of current in vivo studies regarding less accessible endpoints such as for example immunotoxicity or developmental neurotoxicity.

Over recent years, so-called New Approach Methodologies (NAMs) have thus attracted increased attention and importance for regulatory toxicology. The United States Environmental Protection Agency (US EPA 2018) defines NAM as *‘…a broadly descriptive reference to any technology, methodology, approach, or combination thereof that can be used to provide information on chemical hazard and risk assessment that avoids the use of intact animals…*’. One instance of an attempt to replace an animal test with an in vitro test system is the embryonic stem cell test in the area of developmental toxicology (Buesen et al. 2004; Seiler et al. 2006). This stand-alone test was first evaluated for assessing the embryotoxic potential of chemicals as early on as 2004 (Genschow et al. 2004). While its establishment as a regulatory prediction model took several more years, one major outcome was the realization that the use of NAMs in general is greatly improved when used as part of a biologically and toxicologically meaningful testing battery (Marx-Stoelting et al. 2009; Schenk et al. 2010). It should be noted that despite all the potential of such testing batteries a tentative one to one replacement of animal studies is neither practical nor straight forward. The reason is not only the complexity of the endpoints in question but also practical constraints. This was recently exemplified by Landsiedel et al. who pointed out that with the number of different organs and tissues tested during one sub-chronic rodent study, and assuming that 5 NAMs are needed to address the adverse outcomes in any of those organs, it would take decades just to replace this one study. Any regulatory use of NAMs should hence preferably rely on their direct use (Landsiedel et al. 2022).

An example from the field of hepatotoxicity testing is the in vitro toolbox for steatosis that was developed by Luckert et al. (2018) based on the adverse outcome pathway (AOP) concept by Vinken (2015). The authors employed five assays covering relevant key events from the AOP in HepaRG cells after incubation with the test substance Cyproconazole. Concomitantly, transcript and protein marker patterns for the identification of steatotic compounds were established in HepaRG cells (Lichtenstein et al. 2020). The findings were subsequently brought together in a proposed protocol for AOP-based analysis of liver steatosis in vitro (Karaca et al. 2023a).

One promising use for such cell-based systems is their combination with multi-level omics. In conjunction with sufficient biological and mechanistic knowledge, the wealth of information provided by multi-omics data should potentially allow some prediction of substance-induced adversity. That said any such prediction can of course only be reliable within the established limits of such systems such as the lack of a whole organism and incomplete toxicokinetics and restrictions on adequately capturing the effects of long-term exposure (Schmeisser et al. 2023). Regulatory use and trust in cell-based systems will, therefore, strongly rely on how they compare to the outcome of studies based on systemic data (Schmeisser et al. 2023).

Pesticide active substances are a group of compounds with profound in vivo data. Some examples for active substances commonly used in PPPs are the fungicides Cyproconazole, Fluxapyroxad, Azoxystrobin and Thiabendazole, as well as the herbicide Chlorotoluron and the multi-purpose substance 2-Phenylphenol. For these compounds, several short- and long-term studies in rodents have been conducted and multiple adverse effects in target organs like liver or kidneys were observed (see Table 1). Liver steatosis, as one potential adverse health outcome, has been associated with triazole fungicides, such as Cyproconazole, but other active substances such as Azoxystrobin are suspected to interfere with the lipid metabolism as well (Gao et al. 2014; Luckert et al. 2018). Potential modes of action for adverse effects include the activation of nuclear receptors, such as the constitutive androstane receptor (CAR), which has been shown for Cyproconazole and Fluxapyroxad (Marx-Stoelting et al. 2017; Tamura et al. 2013; Zahn et al. 2018). Notably, even when an active substance is considered to be of low acute toxicity, e.g. Chlorotoluron, Thiabendazole and 2-Phenylphenol (EC 2015; US EPA 2002; WHO 1996), they might still exhibit adverse chronic effects (Mizutani et al. 1990; WHO 1996). This is the reason why pesticide active substances and plant protection products (PPP) are assessed extensively before their placing on the market (EC 2009).

**Table 1:** Effects of the test substances as observed in vivo and categorized by Nielsen et al. (2012)

	Liver	Kidneys
Cyproconazole	Cholestasis Hypertrophy Hepatocellular fatty changes Hepatocellular cell degeneration/death Inflammation in the liver Phase I enzyme induction	-
Fluxapyroxad	Hypertrophy Hepatocellular cell degeneration/death Neoplasms Lesions of biliary epithelium Phase I enzyme induction	-
Azoxystrobin	Hypertrophy Hepatocellular cell degeneration/death Inflammation in the liver Lesions of biliary epithelium	-
Chlorotoluron	Hepatocellular fatty changes Inflammation in the liver	Tubular neoplasms
Thiabendazole	Hypertrophy Lesions of biliary epithelium Phase I enzyme induction	Calculi Inflammation Papillary cell degeneration/death Papillary hypertrophy/hyperplasia Tubular cell degeneration/death Tubular fatty changes Tubular hypertrophy/hyperplasia
2-Phenylphenol	Foci of cellular alteration in the liver Neoplasms Phase I enzyme induction Oxidative stress	Calculi Inflammation Oxidative stress Papillary cell degeneration/death Papillary hypertrophy/hyperplasia Pelvis hyperplasia Tubular cell degeneration/death Tubular hypertrophy/hyperplasia

The target organs most frequently affected by pesticide active ingredients are the liver and kidneys (Nielsen et al. 2012). Hence, an in vitro test system aimed at the prediction of pesticide organ toxicity should be able to model effects on these two target organs. One of the best options currently available for hepatotoxicity studies in vitro is the cell line HepaRG (Ashraf et al. 2018). Before their use in toxicological assays, the cells undergo a differentiation process resulting in CYP-dependent activities close to the levels in primary human hepatocytes (Andersson et al. 2012; Hart et al. 2010). They also feature the capability to induce or inhibit a variety of CYP enzymes (Antherieu et al. 2010; Hartman et al. 2020) and the expression of phase II enzymes, membrane transporters and transcription factors (Aninat et al. 2006). Antherieu et al. (2012) demonstrated that HepaRG cells can sustain various types of chemically induced hepatotoxicity following acute and repeated exposure. Hence, HepaRG cells have the potential to replace the use of primary human hepatocytes in the study of acute and chronic effects of xenobiotics in the liver. In 2012, the European Commission Joint Research Centre’s European Union Reference Laboratory for Alternatives to Animal Testing (EURL ECVAM) coordinated a validation study finding differentiated HepaRG cells as a reliable and relevant tool for CYP enzyme activity studies (EURL ECVAM 2012). This led to the proposal of a respective draft test guideline by the OECD in 2019 (OECD 2019). Additionally, as part of the US EPA Tox21 project, HepaRG cells were used for an assay assessing toxicogenomics (Franzosa et al. 2021).

A promising test system for investigations of nephrotoxicity is the tERT1 immortalized renal proximal tubular epithelial cell line RPTEC/tERT1 (further referred to as RPTEC). These non-cancerous cells have been found to closely resemble primary counterparts showing typical morphology and functionality (Shah et al. 2017; Wieser et al. 2008). Aschauer et al. (2015) demonstrated the applicability of RPTEC for investigation of repeated-dose nephrotoxicity using a transcriptomic-based approach. Simon et al. (2014) showed similar toxicological responses of RPTEC and the target tissue to exposure to benzo[*a*]pyrene and cadmium. Conclusively, RPTEC can be a useful tool for toxicological studies.

In the present study, six pesticide active substances were analyzed in two cell lines, namely the liver cell line HepaRG and the kidney cell line RPTEC. Assays were performed following exposure to the highest non-cytotoxic concentration and comprised targeted protein and transcriptomics analysis. Triggered pathways were identified and compared with established results from in vivo experiments.

## Materials and methods

### Materials

All test substances were purchased in analytical grade (purity ≥ 98.0%) from Sigma-Aldrich, Pestanal® (Taufkirchen, Germany): Cyproconazole, CAS no. 94361–06-5, catalog no. 46068, batch no. BCCD4066; Fluxapyroxad, CAS no. 907204–31-3, catalog no. 37047, batch no. BCCF6749; Azoxystrobin, CAS no. 131860–33-8, catalog no. 31697, batch no. BCCF6593; Chlorotoluron, CAS no. 15545–48-9, catalog no. 45400, batch no. BCBW1414; Thiabendazole, CAS no. 148–79-8, catalog no. 45684, batch no. BCBV5436; 2-Phenylphenol, CAS no. 90–43-7, catalog no. 45529, batch no. BCCF1784. William’s E medium, fetal calf serum (FCS) good forte (catalog no. P40-47500, batch no. P131102), recombinant human insulin and l-glutamine were acquired from PAN-Biotech GmbH (Aidenbach, Germany), FCS superior (catalog no. S0615, batch no. 0001659021) from Bio&Sell (Feucht bei Nürnberg, Germany). Dimethyl sulfoxide (DMSO, purity ≥ 99.8%), hydrocortisone-hemisuccinate (HC/HS), hydrocortisone, epidermal growth factor (EGF) and neutral red (NR) were purchased from Sigma-Aldrich (Taufkirchen, Germany). Dulbecco’s modified eagle medium (DMEM) and Ham’s F Nutrition mix were obtained from Gibco® Life Technologies (Karlsruhe, Germany), trypsin–EDTA, Penicillin–Streptomycin and insulin-transferrin-selenium from Capricorn Scientific GmbH (Ebsdorfergrund, Germany).

### Cell culture

#### HepaRG

HepaRG cells were obtained from Biopredic International (Sant Grégoire, France) and kept in 75 cm^2^ flasks under humid conditions at 37 °C and 5% CO_2_. Cells were grown in proliferation medium consisting of William’s E medium with 2 mM l-glutamine, supplemented with 10% FCS good forte, 100 U mL^−1^ penicillin, 100 µg mL^−1^ streptomycin, 0.05% human insulin and 50 µM HC/HS for 2 weeks. Then, HepaRG cells were passaged using trypsin–EDTA solution and seeded in 75 cm^2^ flasks, 6-well, 12-well and 96-well plates at a density of 20 000 cells per cm^2^. Cells in cell culture dishes were maintained in proliferation medium for another 2 weeks before the medium was changed to differentiation medium (i.e., proliferation medium supplemented by 1.7% DMSO) and cells were cultured for another 2 weeks. Thereafter, cells were used in experiments within 4 weeks, while media was changed to treatment media (i.e., proliferation media supplemented by 0.5% DMSO and 2% FCS) 2 days prior to the experiments.

#### RPTEC

The RPTEC cell line was obtained from Evercyte GmbH (Vienna, Austria) and cultivated as previously described (Aschauer et al. 2013; Wieser et al. 2008). Cells were grown in a 1:1 mixture of DMEM and Ham’s F-12 Nutrient Mix, supplemented with 2.5% FCS superior, 100 U mL^−1^ penicillin, 100 µg mL^−1^ streptomycin, 2 mM l-glutamine, 36 ng mL^−1^ hydrocortisone, 10 ng mL^−1^ EGF, 5 µg mL^−1^ insulin, 5 µg mL^−1^ transferrin and 5 ng mL^−1^ selenium. RPTEC were cultivated in 75 cm^2^ flasks until they reached near confluence. Then, cells were passaged using trypsin–EDTA and seeded at 30% density in 75 cm^2^ flasks for further sub-cultivation and 6-well, 12-well and 96-well plates for experiments. To obtain complete differentiation, cells in cell culture dishes were maintained for 14 days before they were used in experiments.

### Test concentrations

All substances were dissolved in DMSO and diluted in the respective medium to a final DMSO concentration of 0.5% before incubation. HepaRG treatment medium and 0.5% DMSO in RPTEC medium served as solvent controls for HepaRG cells and RPTEC, respectively. At least 3 biological replicates, i.e., independent experiments, were performed for each assay.

### Cell viability

Cell viability was investigated with the WST-1 assay (Immunservice, Hamburg, Germany), according to the manufacturer’s protocol and subsequent NR uptake assay according to Repetto et al. (2008). HepaRG cells and RPTEC were seeded in 96-well plates and incubated with the test substances for 72 h. Triton X-100 (0.01%, Thermo Fisher Scientific, Darmstadt, Germany) was used as positive control for reduced cell viability. At the end of the incubation period, 10 µL WST-1 solution was added to each well and incubated for 30 min at 37 °C. The tetrazolium salt WST-1 is metabolized by cellular mitochondrial dehydrogenases of living cells to a formazan derivative, the absorbance of which was measured at 450 nm with an Infinite M200 PRO plate reader (Tecan, Maennedorf, Switzerland). The reading of each well was related to the absorbance value at the reference wavelength of 620 nm, and blank values were subtracted before the relation to the solvent control.

Afterwards the NR uptake assay was performed, where incorporation of NR into lysosomes of viable cells is measured. One day prior to the assay, NR medium was prepared by diluting a 4 mg mL^−1^ NR stock solution in PBS 1:100 with the respective cell culture medium for HepaRG cells and RPTEC, and incubated at 37 °C over night. After the WST-1 measurement, the incubation medium was removed and cells were washed twice with PBS. Subsequently, 100 µL NR medium, previously centrifuged for 10 min at 600 × *g*, was added and incubated for 2 h. Afterwards, cells were washed twice with PBS, and 100 µL destaining solution (49.5:49.5:1 ethanol absolute, distilled water, glacial acetic acid) per well was added. Plates were shaken at 500 rotations min^−1^ for 10 min and fluorescence of NR was measured with an Infinite M200 PRO plate reader (Tecan, Maennedorf, Switzerland) at 530 nm excitation and 645 nm emission. Each reading was subtracted by the blank value and normalized to the solvent control.

### Multiplexed microsphere-based sandwich immunoassays

Marker proteins and protein modifications were analyzed by Signatope GmbH (Tübingen, Germany) with a multiplexed microsphere-based sandwich immunoassay. Cells were seeded in 6-well plates and incubated with the test substances for 36 and 72 h. Protein extraction was performed by adding 250 µL pre-cooled extraction buffer, supplied by the company, to the cells in each well and subsequent incubation for 30 min at 4 °C. Cell lysates were transferred to 1.5 mL reaction tubes and centrifuged for 30 min at 4 °C and 15 000 × *g*. The supernatant was aliquoted in 60 µL batches and stored at -80 °C until shipment. After thawing, aliquots were directly used and not frozen again. Samples were analyzed for 8 proteins and protein modifications, each representing a marker for a certain form of toxicity (Table 2).

**Table 2:** Analyzed proteins or protein modifications and associated cellular functions

Cellular function		Read Out – Protein/ protein modification	
Translation	↑	Phosphorylated elongation factor 4B (S422) (p-elF4B) (Duncan and Hershey 1989; Jackson et al. 2010)	↑
Transcription	↑	Phosphorylated RNA Polymerase II (S2) (p-RNA pol II) (Duncan and Hershey 1989; Muniz et al. 2021)	↑
Protein degradation	↑	Ubiquitin isopeptide branch Lys48 (Ubiquitin k48) (French et al. 2021; Ohtake 2022)	↑
Cell division	↑	Phosphorylated Histone H3 (S10) (p-Histon H3) (Andonegui-Elguera et al. 2022; Nelson et al. 2002)	↑
Oxidative/ heat stress	↑	70-kDa heat shock protein (HSP70) family (Kiang and Tsokos 1998)	↑
Apoptosis	↑	Cleaved poly (ADP-ribose) polymerase (D214) (cleaved PARP) (Oliver et al. 1998)	↑
Autophagy	↑	Microtubule-associated proteins 1A/1B light chain 3B (total LC3B) (Reggiori and Klionsky 2002)	↑
Hypoxia	↑	Hypoxia-inducible factor alpha (HIF 1-alpha) (Lee et al. 2007)	↑

### Quantitative real-time PCR and PCR profiler arrays

RT-qPCR was conducted to ensure well performing RNA for subsequent PCR profiler arrays. Cells were seeded in 12-well plates and incubated with the test substances for 36 h. RNA extraction was performed with the RNA easy Mini Kit (Qiagen, Venlo, Netherlands) according to the manufacturer’s manual. Yield RNA concentration and purity were analyzed with a Nanodrop spectrometer (NanoDrop 2000, Thermo Fischer Scientific, Darmstadt, Germany) and RNA samples were stored at -80 °C until further use. Reverse transcription to cDNA was conducted using the High-Capacity cDNA Reverse Transcription Kit (Applied Biosystems, Waltham, MA, USA) according to the manufacturer’s protocol with a GeneAmp^®^ PCR System 9700 (Applied Biosystems, Darmstadt, Germany) and cDNA samples were stored at – 20 °C. RT-qPCR was performed with Maxima SYBR Green/ROX Master Mix (Thermo Fisher Scientific, Darmstadt, Germany) according to manufacturer’s protocol. In brief, 9 µL master mix, consisting of 5 µL Maxima SYBR Green/ROX qPCR Master Mix, 0.6 µL each of forward and reverse primers (2.5 µM) and 2.8 µL nuclease-free water, was added to each well of a 384-well plate. Primer sequences are shown in Online Resource 1. Subsequently, 20 ng cDNA was added to each well to a final volume of 10 µL and RT-qPCR was performed with an ABI 7900HT Fast Real-Time PCR system instrument (Applied Biosystems, Darmstadt, Germany). In brief, activation took place at 95 °C for 15 min, followed by 40 cycles of 15 s at 95 °C and 60 s at 60 °C, followed by 15 min at 60 °C and default melting curve analysis. Data were processed using 7900 software v241 and Microsoft Excel 2021. Threshold cycle (C_T_) was set to 0.5, melting curve was checked and manual baseline correction was performed for each gene individually. Yield C_T_-values were extracted to Microsoft Excel 2021 and relative gene expression was obtained with the 2^−ΔΔCt^ method according to Livak and Schmittgen (2001). GUSB and HPRT1 served as endogenous control genes for HepaRG cells, GUSB and GAPDH were used for RPTEC. Primer efficiency was tested beforehand according to Schmittgen and Livak (2008). Only RNA samples showing amplification in RT-qPCR were used for further analysis with PCR profiler arrays. For quality control purposes, yield 2^−ΔΔCt^ values from RT-qPCR and PCR profiler arrays were compared and had to be within the same range (Online Resource 1).

For performing the PCR profiler array, cDNA was synthesized from 1 µg RNA using the RT^2^ First Strand Kit (Qiagen, Venlo, Netherlands) according to the manufacturer’s protocol with a GeneAmp® PCR System 9700 (Applied Biosystems, Darmstadt, Germany). Subsequently, the RT^2^ Profiler™ PCR Array *Human Molecular Toxicology Pathway Finder* or *Nephrotoxicity* (Qiagen, Venlo, Netherlands) was conducted with RT^2^ SYBR^®^ Green ROX qPCR Mastermix (Qiagen, Venlo, Netherlands) according to the manufacturer’s protocol. RT-qPCR was performed with an ABI 7900HT Fast Real-Time PCR system instrument (Applied Biosystems, Darmstadt, Germany), where activation of polymerase took place for 10 min at 95 °C, followed by 40 cycles of 15 s at 95 °C and 60 s at 60 °C and default melting curve analysis. Data were analyzed using 7900 software v241 and Excel 2021. C_T_ was set to 0.2, melting curve was checked and manual baseline correction was performed. Yield C_T_-values were extracted and further analyzed.

### Pathway analysis

Further evaluation of PCR array data was performed with functional class scoring methods such as Gene Ontology (GO) enrichment and Kyoto Encyclopedia of Genes and Genomes (KEGG), as well as with the bioinformatics analysis and search tool Ingenuity Pathway Analysis Software (IPA). Following the manufacturer’s instructions, yield C_T_-values were uploaded to the Qiagen Gene Globe Webportal^1^ and analyzed using the standard ΔΔC_T_ method referring to an untreated control. A cut-off C_T_ was set to 35, all 5 built-in housekeeping genes were manually selected as reference genes and their arithmetic mean used for normalization. Means of fold regulation and p-values were calculated and further evaluated with the bioinformatics tools following the protocol provided in Online Resource 2. The processed results from HepaRG cells and RPTEC were used as input data individually, as well as combined. For the combined analysis, duplicate genes that were present on both arrays were removed.

To generate a first overview, the percentage of differentially expressed genes (DEG) per pathway was determined as previously published (Heise et al. 2018). Genes were assorted to pathways as suggested on the manufacturer’s web page.^2^ The percentage of DEG was calculated as number of genes whose expression significantly differed by a fold change of 2, as determined by Student’s *t-*test (p < 0.05), related to the total number of genes in the pathway.

#### GO enrichment and KEGG analysis

The freely available web tools GOrilla^3^ and ShinyGO 0.80^4^ were used for GO enrichment and KEGG analysis, respectively (Eden et al. 2007, 2009; Ge et al. 2020). Detailed protocols are provided in Online Resource 2 together with the R code for determining DEG and background genes (see Data availability), which was adapted from Feiertag et al. (2023).

#### Ingenuity pathway analysis

In addition to GO enrichment and KEGG analysis, further evaluation of PCR array data was performed with the bioinformatics analysis and search tool IPA (Qiagen, Hilden, Germany, analysis date: Nov. 2023) as previously published (Karaca et al. 2023b). IPA is a commercial bioinformatics tool for analyzing RNA data, predicting pathway activation and functional interrelations using a curated pathway database. Using Fisher’s exact test, IPA identifies overrepresented pathways by measuring significant overlaps between user-provided gene lists and predefined gene sets. Means of fold regulation and *p*-values were uploaded to IPA following the protocol provided in Online Resource 2. Cut-off was set to – 1.5 and + 1.5 for fold regulation and 0.05 for the *p*-value. Fold regulation represents fold change results in a biologically meaningful way. In case the fold change is greater than 1, the fold regulation is equal to the fold change. For fold change values less than 1, the fold regulation is the negative inverse of the fold change. No further filtering was applied and an IPA core analysis was run. One Excel spread sheet per substance was obtained including all predicted diseases or functions annotations, the associated categories, the p-value of overlap as well as the number and names of the DEG found in the respective annotation (Online Resource 3). Predicted effects on other organs than the liver or the kidneys, such as heart or lungs, were discarded. For further comparison with in vivo data only the categories were used, combined with the p-value of the annotation, which was the highest.

### Comparison with animal studies

The data obtained from targeted protein and transcriptomics analyses were compared with known in vivo observations from Draft Assessment Reports (DARs) of the pesticide active substances required for pesticide legislation. To facilitate the comparison of the data, the in vitro data was transformed into a more comprehensible form by applying evaluation matrices as shown in Table 3.

**Table 3:** Evaluation matrices for analysis of targeted protein and transcriptomics further analyzed with Ingenuity Pathway Analysis software (IPA)

	Protein		IPA	
Evidence	Condition	Symbol	*p*-value	Symbol
Very strong	≥ 2 conditions > 200%	+ + +	≤ 0.0005	+ + +
Strong	1 condition > 200%/ 2 conditions 150–200%	+ +	≤ 0.005	+ +
Medium	1 condition 150–200%	+	≤ 0.05	+

The in vivo effects attributed to the pesticide active substances were taken from the publication by Nielsen et al. (2012). Additionally, the DARs of the two substances not reported in Nielsen et al*.* were analyzed and assigned accordingly. All in vivo effects identified by the authors for liver and kidneys can be found in Online Resource 1. Based on expert knowledge, descriptions of in vitro outcomes were combined with in vivo observations (see Tables 4 and 5).

**Table 4:** Combination of prediction from marker proteins with potential in vivo observations as categorized by Nielsen et al. (2012)

Cellular function		In vivo liver	In vivo kidneys
Translation	↑	Neoplasms	Tubular neoplasms
		Foci of cellular alteration in the liver	Tubular hypertrophy/hyperplasia
		Hypertrophy	
Cell division	↑	Neoplasms	Tubular neoplasms
		Foci of cellular alteration in the liver	Tubular hypertrophy/hyperplasia
Oxidative/ heat stress	↑	Oxidative stress	Oxidative stress
Autophagy	↑	Hepatocellular cell degeneration/death	Tubular cell degeneration/death
Apoptosis	↑	Hepatocellular cell degeneration/death	Tubular cell degeneration/death
Transcription	↑	Neoplasms	Tubular neoplasms
		Foci of cellular alteration in the liver	Tubular hypertrophy/hyperplasia
		Hypertrophy	
Protein degradation	↑	–	–
Hypoxia	↑	–	–

**Table 5:** Combination of prediction from Ingenuity Pathway Analysis (IPA) of transcriptomics data with potential in vivo observations as categorized by Nielsen et al. (2012)

IPA prediction	In vivo liver/ kidneys
Hepatocellular carcinoma, Liver Hyperplasia/Hyperproliferation	Neoplasms
Liver Cholestasis	Cholestasis
Liver Cholestasis, Liver Inflammation/Hepatitis	Cholestasis
	Inflammation in the liver
Liver Hyperplasia/Hyperproliferation	Neoplasms
Liver Inflammation/Hepatitis	Inflammation in the liver
Liver Inflammation/Hepatitis, Liver Steatosis	Hepatocellular fatty changes
	Inflammation in the liver
Liver Necrosis/Cell Death	Hepatocellular cell degeneration/death
Liver Proliferation	Foci of cellular alteration in the liver
	Neoplasms
Liver Steatosis	Hepatocellular fatty changes
Glomerular Injury	Glomerular cell degeneration
Glomerular Injury, Renal Inflammation, Renal Nephritis	Glomerular cell degeneration
	Glomerular inflammation
	Inflammation
Renal Damage, Renal Tubule Injury	Tubular cell degeneration/death
Renal Inflammation, Renal Nephritis	Inflammation
Renal Necrosis/Cell Death	Tubular cell degeneration/death
	Glomerular cell degeneration
	Papillary cell degeneration/death
Renal Proliferation	Tubular neoplasms

Based on the combination of the in vitro and the in vivo data, it was possible to draw conclusions on the concordance of the predictions. In order to establish optimized thresholds for regarding an effect as in vitro positive, the analyses were performed by considering at least medium effects, strong and very strong effects, or very strong effects only (see Table 3) and comparing these to the corresponding in vivo effect. In case multiple in vitro predictors were connected to the same in vivo observation, a positive prediction from one was sufficient to be considered in vitro positive. For protein analyses, the comparison was performed for the data from HepaRG cells and RPTEC individually, as well as combined, where a positive prediction from one of the cell lines was considered sufficient and compared to hepatotoxic and nephrotoxic in vivo effects. For the gene transcription analysis, the categories obtained by IPA were compared to in vivo observations from DARs. A further evaluation integrating protein and transcriptional data was conducted, wherein a positive result from either data type was sufficient to classify a sample as in vitro positive. Online Resource 1 shows the combination of the results in detail. The percentage of concordance between in vitro prediction and in vivo observation was calculated. Indicative concordance was defined as percentage of in vivo positive observations that were predicted to be positive by the in vitro test system.

### Statistical analysis

Statistical analysis was performed using R 4.2.1 and RStudio 2023.09.1 + 494. Data evaluation was done with Microsoft Excel 2021.

All experiments were performed in at least three independent biological replicates. Technical replicates, when applicable, were averaged and subsequently mean and standard deviation values were calculated from biological replicates. For targeted protein analysis, statistical significance was calculated with bootstrap technique using R package boot (Canty and Ripley 2016; Davidson and Hinkley 1997) to account for the high variability that results when the protein expression is affected. Data visualization was done using ggplot2 package (Wickham 2016). Calculation of statistical significance of altered gene transcription was performed using Student’s *t*-test, and R package ComplexHeatmaps was used for data visualization (Gu 2022). All R scripts can be found using the link provided in the Data availability section.

## Results

### Impairment of cell viability

Each substance was tested for its effect on the viability of HepaRG cells and RPTEC. Based on these results, the highest non-cytotoxic concentration was determined and employed in further experiments together with a second concentration (i.e., 0.33 × highest non-cytotoxic concentration). For HepaRG cells, published data were used as a starting point for cytotoxicity testing and confirmed with WST-1 and NR uptake assays. The highest non-cytotoxic concentration, defined as the concentration determining a cell viability greater than 80%, is shown in Table 6.

**Table 6:** References for non-cytotoxic concentrations in HepaRG cells and selected concentration of test substances for further testing (cell viability > 80% after 72 h exposure determined with WST-1 and NR uptake assays in technical triplicates)

Test substance	from literature (HepaRG)		HepaRG (n = 2)	RPTEC (n ≥ 3)
Cyproconazole	300 µM 685 µM	(Lichtenstein et al. 2021)^a^ (Zahn et al. 2018)^b^	120 µM	300 µM
Fluxapyroxad	150 µM 164 µM	(Lichtenstein et al. 2021)^a^ (Zahn et al. 2018)^b^	60 µM	30 µM
Azoxystrobin	75 µM 20 µM	(Lichtenstein et al. 2021)^a^ (Zahn et al. 2018)^b^	45 µM	3 µM
Chlorotoluron	-	-	750 µM	900 µM
Thiabendazole	50 µM	(Mennecozzi et al. 2012)^c^	300 µM	900 µM
2-Phenylphenol	270 µM	(Ozawa et al. 2000)^d^	240 µM	210 µM

^a^EC50, HepaRG, 72 h, WST-1 assay

^b^EC80, HepaRG, 24 h, NR uptake assay

^c^no observed cell loss, HepaRG, 72 h, cell count staining

^d^IC50, HepG2, 48 h, crystal violet staining

For RPTEC, a relatively new cell line, little data was available. At least 3 biological replicates were performed in technical triplicates to determine the highest non-cytotoxic concentrations (Table 6). The bar graphs in Online Resource 4 depict the concentration-dependent course of all tested concentrations per substance limited by solubility. Online Resource 1 provides a table with calculated approximations of substance concentrations in the target organ at LOAEL or NOEAL level based on in vivo toxicokinetic results from DARs. These approximations can be compared with the selected in vitro concentrations based on cytotoxicity experiments.

### Effects on marker proteins

**Fig. 1:**
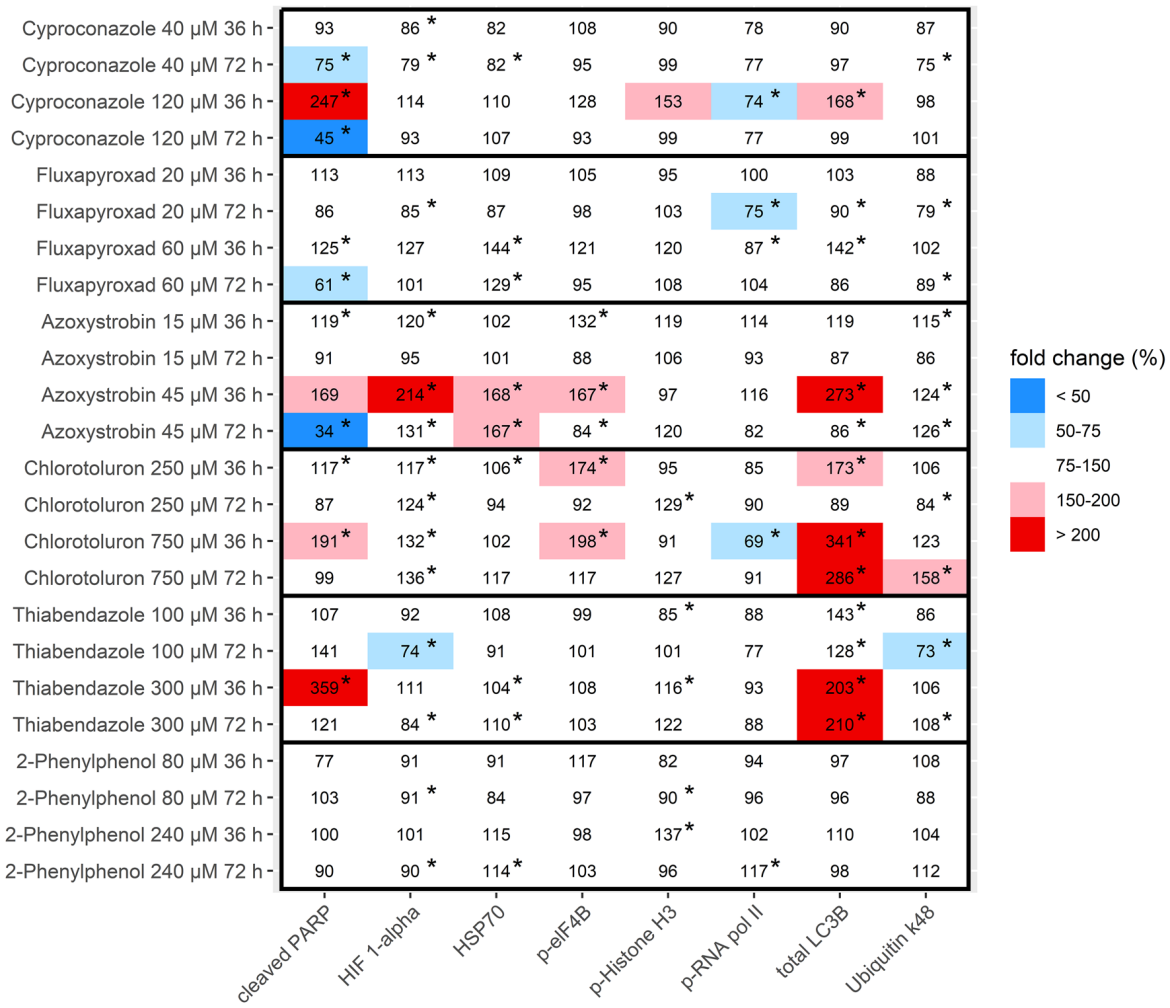
Effects on protein abundance and protein modification of key proteins observed in HepaRG cells after 36 and 72 h of incubation with the test substances using a multiplexed microsphere-based sandwich immunoassay panel. Results are shown as means of 3 independent experiments, normalized to solvent controls. Statistical differences to the solvent control were calculated with bootstrapping (*p < 0.05)

**Fig. 2:**
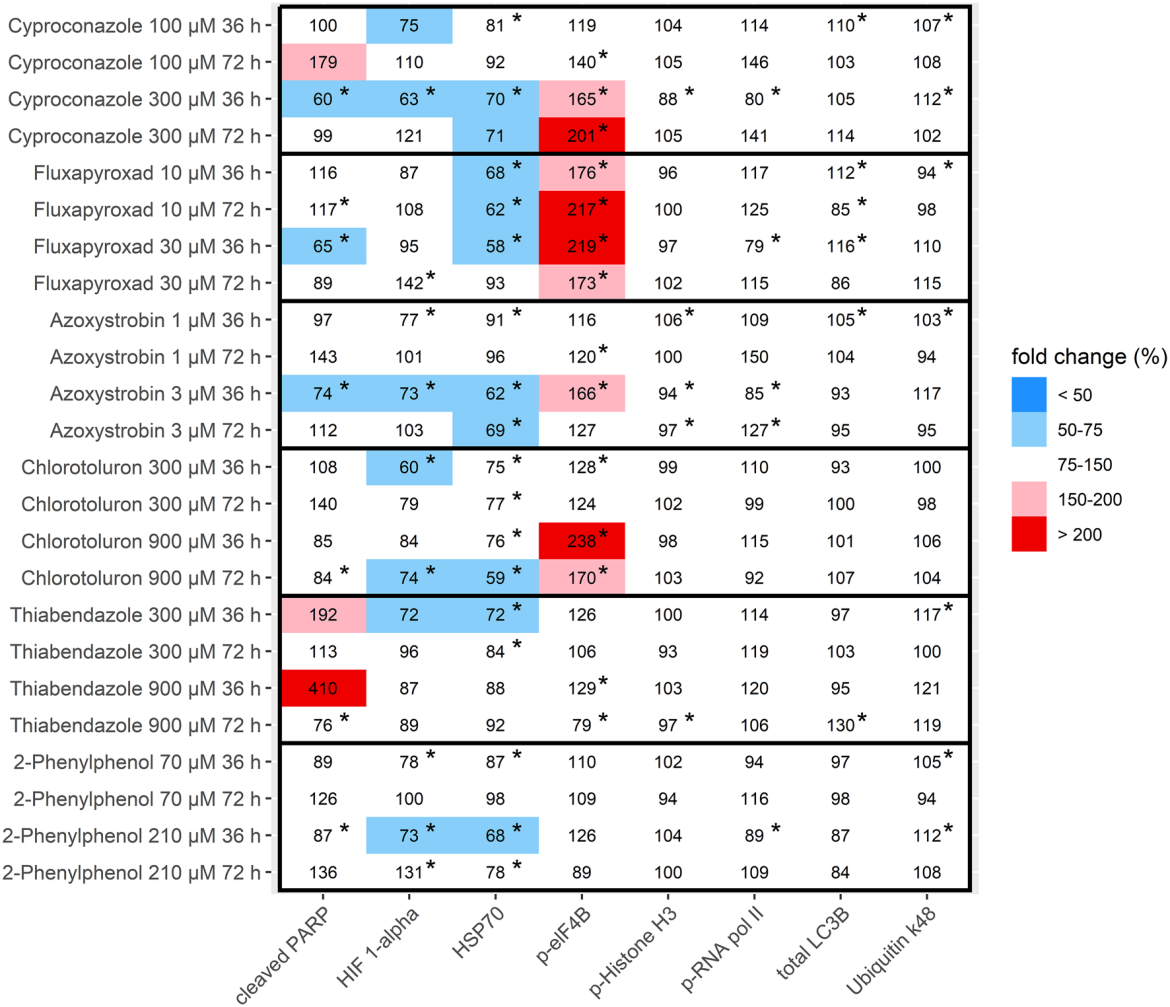
Effects on protein abundance and protein modification of key proteins in RPTEC after 36 and 72 h of incubation with the test substances using a multiplexed microsphere-based sandwich immunoassay panel. Results are shown as means of 3 independent experiments, normalized to solvent controls. Statistical differences to the solvent control were calculated with bootstrapping (*p < 0.05)

The result from multiplex microsphere-based sandwich immunoassays of treated HepaRG cells and RPTEC are shown in Figs. 1 and 2, respectively. In HepaRG cells, incubation with the highest non-cytotoxic concentrations of Azoxystrobin, Chlorotoluron and Thiabendazole increased the expression of total LC3B, an indicator of autophagy, after 36 h (all three compounds) and 72 h (Chlorotoluron and Thiabendazole). Strong effects were observed on cleaved PARP, an indicator of apoptosis, after 36 h of incubation with 120 µM Cyproconazole (247 ± 147%) and 300 µM Thiabendazole (359 ± 204%). However, after 72 h incubation with 120 µM Cyproconazole, the level of cleaved PARP was strongly reduced. Expression of HIF 1-alpha, an indicator of hypoxia, was significantly increased after 36 h incubation with 45 µM Azoxystrobin (214 ± 24%). Fluxapyroxad and 2-Phenylphenol did not significantly increase the expression of any of the protein analytes.

In RPTEC, the abundance of p-elF4B, involved in eukaryotic translation initiation, was increased after 36 and 72 h incubation with 300 µM Cyproconazole (165 ± 45% and 201 ± 51%, respectively), all conditions of Fluxapyroxad, incubation with 3 µM Azoxystrobin for 36 h (166 ± 56%) and incubation with 900 µM Chlorotoluron for 36 and 72 h (238 ± 59% and 170 ± 44%, respectively). Thiabendazole exposure for 36 h resulted in an increase of cleaved PARP at both tested concentrations. Due to the high standard deviation, these results were not statistically significant.

Comparing the results from HepaRG cells and RPTEC, fewer effects were observed in RPTEC than in HepaRG cells. Effects of Azoxystrobin and Chlorotoluron on p-elF4B were observed in both cell lines, as well as increased levels of cleaved PARP after Thiabendazole exposure; yet these results were only significant in HepaRG cells. 2-Phenylphenol did not increase the expression of any of the tested proteins in either cell line, while Fluxapyroxad only affected p-elF4B in RPTEC.

A graphical representation of all data points from HepaRG and RPTEC including means and standard deviations can be found in Online Resource 4.

### Changes at the gene transcription level

**Fig. 3:**
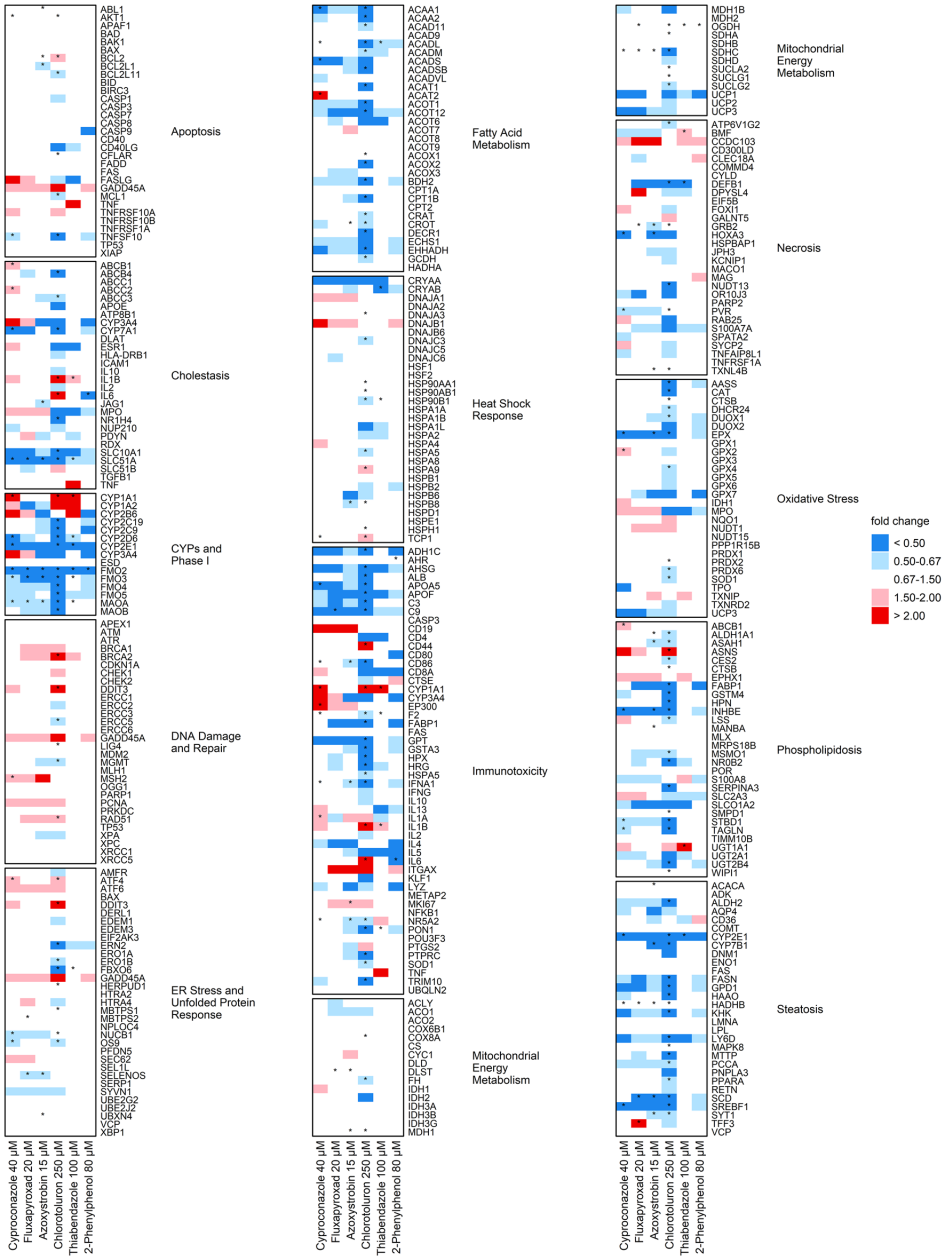
Relative quantities of mRNA transcript levels observed after 36 h exposure of HepaRG cells to non-cytotoxic concentrations of the test substances using the Human Molecular Toxicology Pathway Finder RT^2^ Profiler™ PCR Array. Data evaluation was performed using the 2^−∆∆^^Ct^ method, according to Livak and Schmittgen (2001). All target genes were normalized to 5 housekeeping genes. Results are shown as mean of 3 biological replicates and statistical analysis was performed by one sample Student’s *t*-test (*p < 0.05)

**Fig. 4:**
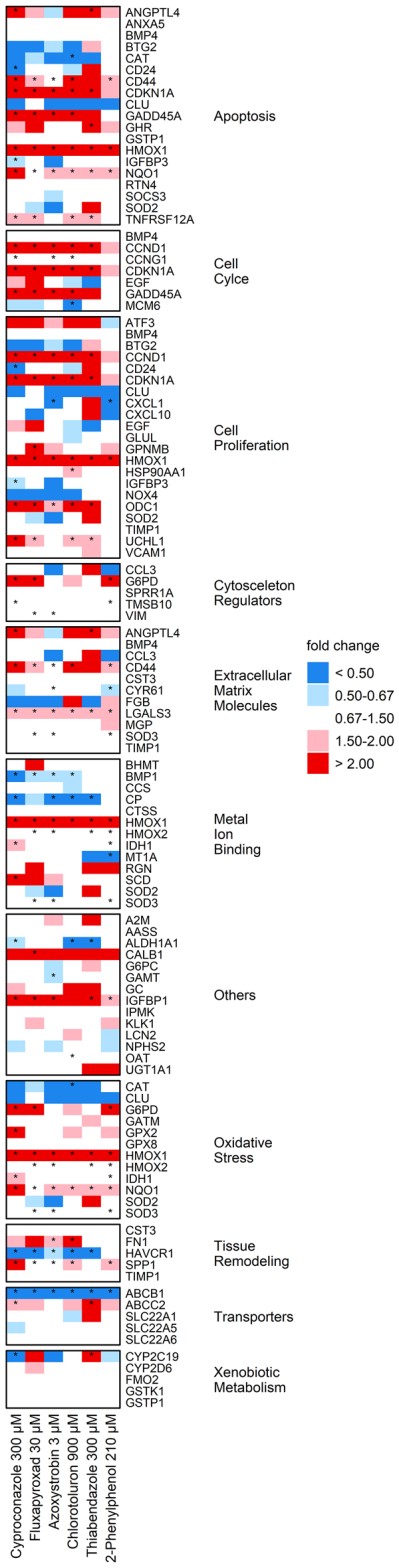
Relative quantities of mRNA transcript levels observed after 36 h exposure of RPTEC to non-cytotoxic concentrations of the test substances using the Human Nephrotoxicity RT^2^ Profiler™ PCR Array. Data evaluation was performed using the 2^−∆∆^^Ct^ method, according to Livak and Schmittgen (2001). All target genes were normalized to 5 housekeeping genes. Results are shown as mean of 3 biological replicates and statistical analysis was performed by one sample Student’s *t*-test (*p < 0.05)

Changes at the protein level are often preceded by changes at the gene expression level. These were analyzed by RT^2^ Profiler™ PCR arrays. Figures 3 and 4 show the results from HepaRG cells and RPTEC, respectively. The genes included in the array were assigned to certain pathways according to the information provided on the manufacturer’s web page. For data interpretation, the percentage of DEG was calculated. In HepaRG cells, most DEG were observed following the exposure to Chlorotoluron. Overall, genes categorized as CYPs and phase I were predominantly affected. Cyproconazole and Chlorotoluron exerted effects on genes associated with fatty acid metabolism (10 and 55%, respectively). Of all steatosis-associated genes, 47% were altered by Chlorotoluron. With regards to individual genes, the strongest increase was observed for *CYP1A1* and *CYP1A2*, both in the group of CYPs and phase I, after exposure to Chlorotoluron (479-fold and 57-fold, respectively) and Thiabendazole (330-fold and 215-fold, respectively).

In RPTEC, the cluster encompassing most of the DEG was that associated with regulation of the cell cycle. Here, Cyproconazole, Fluxapyroxad, Azoxystrobin, and Chlorotoluron affected the expression of over 40% of the associated genes. Genes associated with apoptosis were altered following the exposure to all substances, particularly Cyproconazole and Chlorotoluron (47 and 37%, respectively). Cyproconazole additionally showed pronounced effects on genes encoding for extracellular matrix and tissue remodeling molecules (27 and 40%, respectively). All substances affected about 20% of all genes contained in the group of genes related to cell proliferation. Cyproconazole, Chlorotoluron and 2-Phenylphenol affected 25% of all oxidative stress-associated genes. In comparison to HepaRG cells, where CYPs and phase I was the most impacted group, in RPTEC only one of the DEG established for any of the substances belonged to the group of xenobiotic metabolism. At the level of individual genes, HMOX1, a nephrotoxicity marker, was induced over twofold after incubation with all substances, but highest for Cyproconazole (eightfold). Of all genes, the strongest induction was observed for *IGFBP1*, a member of the insulin-like growth factor-binding protein family, which was increased 53-fold by incubation with Cyproconazole and over 52-fold after incubation with Chlorotoluron.

A graphical representation of all data points including means and standard deviations can be found in Online Resource 4 for HepaRG and RPTEC results.

#### Data analysis with GO enrichment and KEGG analysis

Gene expression results were analyzed with GO enrichment and KEGG analysis. All effects obtained in the analyses can be found in Online Resource 3.

The GO enrichment analysis of HepaRG DEG from the incubation with Cyproconazole pointed at changes in *secondary* and *xenobiotic metabolic processes*, and the combined analysis additionally resulted in significant enrichment of *response to estrogen*. DEG modulated by the exposure to Chlorotoluron were involved in 16 ontologies including *metabolic, biosynthetic,* and *catabolic processes*, with *lipid metabolic process* and *organic hydroxyl compound metabolic process* being the most statistically supported (i.e., p-value: 9.2 × 10^–8^ and 7.7 × 10^–7^, respectively). In RPTEC, *nucleic acid metabolic process* was the only significantly enriched GO term for Chlorotoluron, while the combined analysis revealed a total of 23. Analysis of DEG from incubation with Thiabendazole resulted, among others, in hits for *xenobiotic, terpenoid,* and *isoprenoid metabolic process* in HepaRG and combined results. Although analysis of DEG from incubation with 2-Phenylphenol did not result in significantly enriched GO terms from the HepaRG or the RPTEC data; the combined data set showed 5 enriched terms with *NADP metabolic process* and *myeloid leukocyte migration* having the lowest p-values (6.9 × 10^–4^, both).

For KEGG analysis, the HepaRG data set for Fluxapyroxad and Chlorotoluron showed enrichment of *drug metabolism-cytochrome P450*, as well as *taurine and hypotaurine metabolism* (Fluxapyroxad) and *metabolic pathways* (Chlorotoluron). Thiabendazole data revealed enrichment of *steroid hormone biosynthesis*, *metabolism of xenobiotics by cytochrome P450* and *chemical carcinogenesis-DNA adducts*. RPTEC data set for Azoxystrobin and Chlorotoluron showed multiple cancer-related pathways. The combined data set only resulted in few pathways: *hepatocellular carcinoma* for Azoxystrobin, *metabolic pathways* for Chlorotoluron and *mineral absorption* for 2-Phenylphenol. All other analyses did not result in any significant enrichment.

#### Data analysis with ingenuity pathway analysis software

**Fig. 5:**
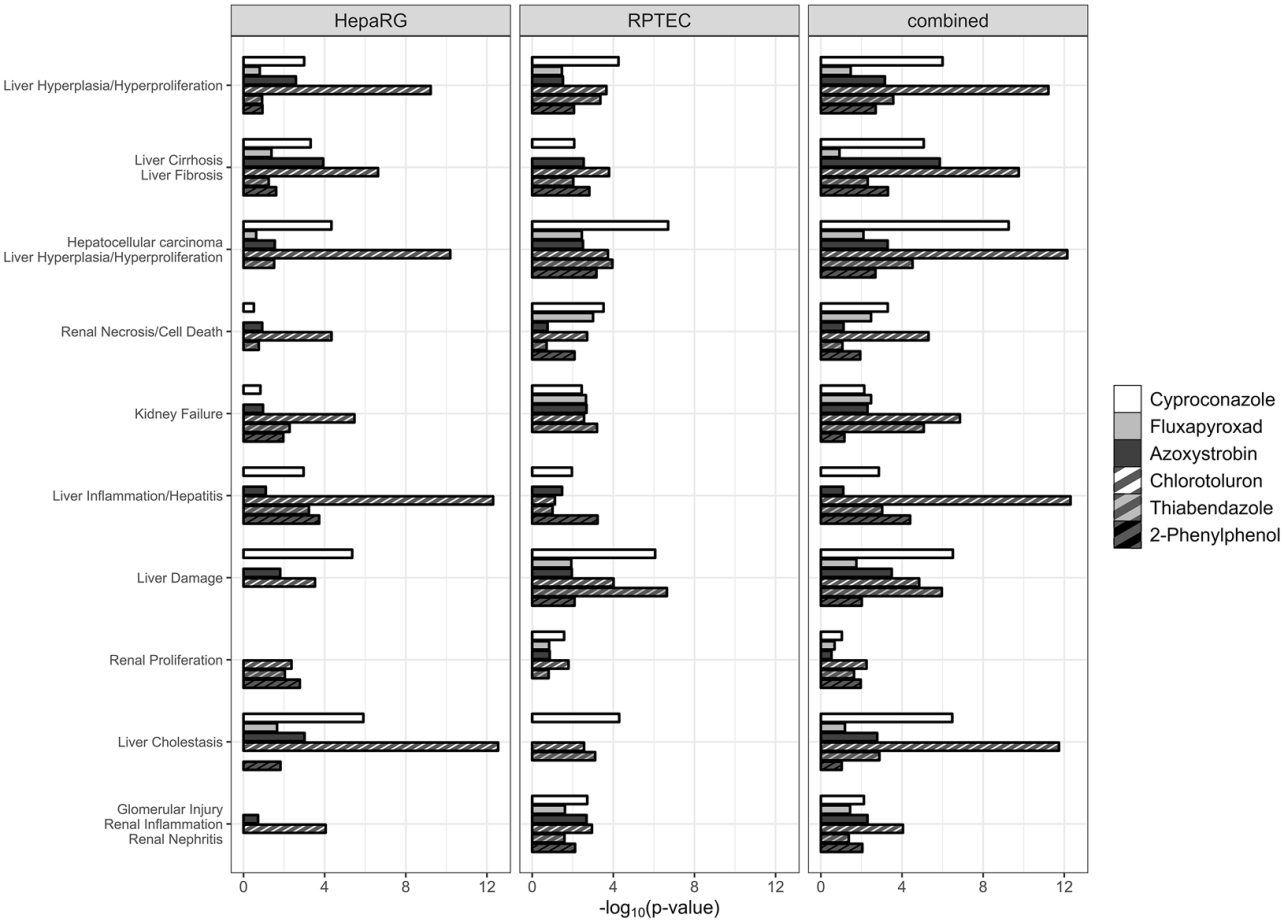
Results obtained by analysis of transcriptomics data with Qiagen Ingenuity Pathway Analysis. The 10 categories most affected are represented. The x-axis shows the -log_10_ value of the p-value obtained for the respective effect

Gene expression data were further analyzed with the IPA software. In total 32 different categories of diseases or functions were predicted. Figure 5 shows the ten most frequently resulting categories. *Liver Hyperplasia/Hyperproliferation* is the only common category across all cell lines and substances. The statistical confidence of the pathway analysis was strongest for Chlorotoluron, which also induced most DEG. Comparing the three methodologies of input data, lower p-values were observed for HepaRG and combined analysis and most categories of diseases or functions were predicted by the combined analysis. Evidently, effects on the kidney were predicted from the input data from liver cells and vice versa.

### Comparison with animal studies

In a final step, the data acquired from targeted protein and transcriptomics analyses were compared with known in vivo observations. Given that the comparison focused on aligning the responses from human cell lines with whole animal data, the analysis focused on the extent to which the omics-responses were indicative of the respective biological response in vivo (indicative concordance). To establish an optimized threshold for the evaluation of in vitro predictions, the in vitro data were transformed by applying evaluation matrices as shown in Table 3. Based on that, activated key proteins and thus cellular functions were identified for each substance from targeted protein analyses. For the evaluation of gene transcripts, the p-values for the categories obtained by IPA were considered. Indicative concordance with known in vivo results is shown in Table 7.

**Table 7:** Indicative concordance (%) for a) targeted protein analysis, b) Ingenuity Pathway Analysis of transcriptomics data and c) combined analysis in HepaRG cells, RPTEC and combined evaluation compared to in vivo observations

			Medium ( +)	Strong (+ +)	Very strong (+ + +)
a)	Protein	HepaRG	18	18	0
		RPTEC	20	17	0
		combined	47	41	18
b)	mRNA	HepaRG	55	55	27
		RPTEC	63	0	0
		combined	76	47	24
c)	combined	combined	88	75	25

For the protein analysis, the indicative concordance ranged from 18 to 47% for the single cell lines and their combination, respectively. In contrast to the results from targeted protein analyses, the indicative concordance for the transcriptomic response was much stronger with greatest values of 55, 63 and 76% for the single cell lines and their combination, respectively. Likewise, for those cases where no effect was seen in vivo, no adverse indications were seen in vitro in 80, 91 and 78% of cases, respectively. For protein analysis, this value ranged from 78 to 86% and was 50% for the combined analysis of protein and transcriptional data. It should be noted, however, that these values decreased when the evaluation criteria were less strict (medium or strong instead of very strong).

## Discussion

In the present study, the pathways triggered by non-cytotoxic concentrations of six pesticide active substances were examined, employing targeted protein and transcriptomics analyses in the liver cell line HepaRG and the kidney cell line RPTEC. Utilizing evaluation matrices and prediction software tools, the observed cellular responses were interpreted and compared with outcomes from established in vivo experiments, in order to assess the relevance of our in vitro model systems in predicting the impact of pesticide exposure on human hepatic and renal cellular function. The primary emphasis of this investigation did not lie in delineating discrete effects attributable to individual substances; rather, it centered on discerning the predictive capacity of the system and serving as a case study to highlight the current challenges in the regulatory adoption of NAMs.

When targeted protein data were used to predict in vivo impacts in rodents, the best result was achieved by the combined analysis and setting the evaluation criteria to medium effects (47%). Regarding the indicative concordance based on transcriptional data, medium effects in HepaRG cells seemed the most promising resulting in a 55% match. This is notable given the systemic as well as species differences between the corresponding test systems. It also highlights that the “gold standard”, i.e., the reference standard used for comparison, is in fact not necessarily indisputable (Trevethan 2017). Various studies pointed at the shortcomings of traditional animal studies, such as interspecies concordance, poor reproducibility and unsatisfactory extrapolation to humans (Goodman 2018; Karmaus et al. 2022; Luijten et al. 2020; Ly Pham et al. 2020; Smirnova et al. 2018; Wang and Gray 2015). One example illustrating the difficulties in extrapolating data from rodents to humans is the question whether Cyproconazole causes neoplasms in the liver. Here, animal studies with CD-1 mice showed statistically significant positive trends for hepatocellular adenomas and combined tumors in male mice (EFSA 2010; Hester et al. 2012). Ensuing studies identified CAR activation by Cyproconazole as the underlying Mode of Action (MoA) (Peffer et al. 2007). Marx-Stoelting et al. (2017) investigated effects of Cyproconazole in mice with humanized CAR and PXR and demonstrated increased sensitivity of rodents to CAR agonist-induced effects, compared to humanized mice. In line with these observations the Joint FAO/WHO Meeting on Pesticide Residues (JMPR) concluded that Cyproconazole is unlikely to pose a carcinogenic risk to humans (JMPR 2010). Likewise, Cyproconazole was not considered to cause neoplasms in the liver when analyzed for this study. However, such detailed analysis of a substance’s MoA is scarce.

Another important factor impeding the comparison of in vitro and in vivo data are the different ontologies. The need for harmonized ontologies and reporting formats of in vivo data has been expressed by many researchers in the field of in silico toxicology and has been addressed in multiple projects (Hardy et al. 2012; Sanz et al. 2017). For example, uncertainty arises as to the reason if and why an effect for a particular organ is possibly not reported. Depending on the case and study in question, this might be because absent effects were simply not explicitly reported as negative, or because other organ toxicities occurred at lower doses and hence data for the remaining organs were omitted or not assessed, or because the focus of the study was another organ (Smirnova et al. 2018). While this does not pose a problem for when such studies are used for risk assessment, it does affect the comparison with in vitro results. Another major obstacle is the retrospective conclusive combination of large and comprehensive sets of mechanistic data in vitro with systemic and histopathological observations in vivo. This issue has recently been picked up by on-going European ONTOX project^5^ (“ontology-driven and artificial intelligence-based repeated dose toxicity testing of chemicals for next generation risk assessment “) and has led the consortium to reverse the strategy and build NAMs to predict systemic repeated dose toxicity effects to enable human risk assessment when combined with exposure assessment (Vinken et al. 2021). A recent publication by Jiang et al. (2023) as part of the ONTOX project identified transcriptomic signatures of drug-induced intrahepatic cholestasis with potential future use as prediction model. However, not all pathologies have been analyzed so far, and those that have were often only studied for a limited number of chemicals, limiting their transferability. Hence, this study relied on the use of computational tools such as IPA, GO enrichment and KEGG analysis, to draw functional conclusions from transcriptomics data. While IPA results in categorized diseases or functions annotations, KEGG and GO analyses display enriched ontologies. Therefore, while KEGG and GO results were too ambiguous to be related to distinct in vivo observations, it was feasible to combine IPA results with in vivo observations. It is noteworthy that even though GO enrichment and KEGG analysis seem fairly similar, the results varied widely between the predictions from the various software tools. Soh et al. (2010) analyzed consistency, comprehensiveness, and compatibility of pathway databases and made several crucial findings such as the inconsistency of associated genes across different databases pertaining to the same biological pathway. Furthermore, common biological pathways shared across different databases were frequently labeled with names that provided limited indication of their interrelationships. Chen et al. (2023) demonstrated that using the same gene list with different analysis methods may result in non-concordant overrepresented, enriched or perturbed pathways. Taken together, these considerations may explain the divergent findings from the different transcriptomics analyses in the present study. Additionally, these findings underscore the challenges associated with integrating pathway data from diverse sources and emphasize the need for standardized and cohesive representation of biological pathways in databases.

Compared to the transcriptomic data, protein analyses from HepaRG cells and RPTEC cells resulted in a comparatively low indicative concordance. This challenges the notion that protein analysis may be superior in prediction (Wu et al. 2023). One likely explanation is that proteins often reflect molecular functions and adverse effects more accurately, and diseases frequently involve dysregulated post-translational modifications, which are challenging to detect and may be poorly correlated with mRNA levels (Kannaiyan and Mahadevan 2018; Kelly et al. 2010; Zhao et al. 2020). However, due to the relatively low number of protein markers as compared to the number of mRNA markers, the targeted transcriptomics analysis is associated with a higher likelihood of finding a match. In the gene transcription analysis with ensuing IPA evaluation, 370 genes were analysed for HepaRG. In contrast, the protein analysis conducted in this study focussed on 8 proteins or modifications, each indicative of a particular cellular function, that were analysed at two time points after incubation of cells with two concentrations of the test substances. Consequently, a cellular response to a stressor over time can be observed, such as the different levels of cleaved PARP after 36 h and 72 h of incubation with Cyproconazole in HepaRG cells. While elevated levels of this apoptosis indicator were noted after 36 h, reduced levels were observed after 72 h. Possible explanations for this include a cellular feedback mechanism or an advanced stage of apoptosis.

Another central observation is that combination of cell lines and methods significantly increases indicative concordance (up to 88%). In the case of targeted protein analysis, combination of results led to an overall value of 47%, compared to approximately 20% for each cell line. Similar trends were observed for transcriptomic data with 76% indicative concordance for combined results, albeit decreasing the cases where an in vivo negative effect corresponded to no adverse indication seen in vitro*,* as the total number of positive in vitro effects was increased. Nonetheless, the idea that including omics data in regulatory process will unreasonably increase positive findings and lead to overprotectiveness can be challenged as strengthening the evaluation criteria lead to a reversion of this trend. The shortcomings of stand-alone in vitro tests to replace animal experiments have long been known. For example, single tests do not cover all possible outcomes of interest or all modes of action possibly causing a toxicological effect (Hartung et al. 2013; Rovida et al. 2015). In the present study, reported in vivo effects such as lesions of biliary epithelium or inflammation of the liver may not be fully represented by a single hepatic cell line. Hence, regulatory toxicologists strive to implement so-called integrated testing strategies (ITS) (Caloni et al. 2022). Results from projects in the fields of embryonic, developmental and reproductive, or acute oral toxicity have shown that test batteries increase the predictive value over individual assays (Piersma et al. 2013; Prieto et al. 2013; Sogorb et al. 2014). To share these novel methodologies in ITS for safety evaluations in the regulatory context, the OECD Integrated Approaches for Testing and Assessment (IATA) Case Studies Project offers a platform where comprehensive information on case studies, such as consideration documents capturing learnings and lessons from the review experience, can be found.^6^

While this publication’s scope did not extend to establishing a conclusive ITS for liver and kidney toxicity, it serves as a valuable starting point for future analyses in this direction and offers ongoing assistance and insights. Moving forward, it could prove beneficial when exploring testing protocols that integrate protein and transcriptomics analyses, enhancing the comprehensiveness of safety evaluations in this domain.

## Supplementary Information

Below is the link to the electronic supplementary material.

Supplementary file1 (PDF 828 KB)

Supplementary file2 (PDF 250 KB)

Supplementary file3 (XLSX 295 KB)

Supplementary file4 (PDF 4573 KB)

## Data Availability

The data sets generated during the current study are available in the Jochum-et-al-2024 GitHub repository, https://github.com/KristinaJochum/Jochum-et-al-2024.

## Footnotes

1 https://geneglobe.qiagen.com/de/analyze, accessed last: 23.02.2024.

2 https://geneglobe.qiagen.com/us/product-groups/rt2-profiler-pcr-arrays, accessed last: 23.02.2024.

3 https://cbl-gorilla.cs.technion.ac.il/, accessed last: 16.01.2024.

4 http://bioinformatics.sdstate.edu/go/, accessed last: 16.01.2024.

5 https://ontox-project.eu/, accessed last 26.02.2024.

6 https://www.oecd.org/chemicalsafety/risk-assessment/iata/, accessed last: 26.04.2024.
