# Supplementary material for: Comparative case study on NAMs: towards enhancing specific target organ toxicity analysis
Source: Arch Toxicol. 2024 Aug 29;98(11):3641–58. doi: 10.1007/s00204-024-03839-7 (PMC11489238; doi:10.1007/s00204-024-03839-7)
Supplement: Supplementary file 1 — Supplementary file1 (PDF 828 KB) [file 204_2024_3839_MOESM1_ESM.pdf]

Comparative Case Study on NAMs: Towards Enhancing Specific Target Organ Toxicity Analysis

Archives of Toxicology

Kristina Jochum[1], Andrea Miccoli[1,2,5], Cornelia Sommersdorf[3], Oliver Poetz[3,4], Albert Braeuning[5], Tewes Tralau[1], Philip Marx-Stoelting[1]

[1] German Federal Institute for Risk Assessment, Department of Pesticides Safety, Berlin, Germany

[2] National Research Council, Institute for Marine Biological Resources and Biotechnology (IRBIM), Ancona, Italy

[3] Signatope GmbH, Tübingen, Germany

[4] NMI Natural and Medical Sciences Institute at the University of Tübingen, Reutlingen, Germany

[5] German Federal Institute for Risk Assessment, Department of Food Safety, Berlin, Germany

philip.marx-stoelting@bfr.bund.de

Online Resource 1 Compilation of supplementary tables, including information relevant to gene transcription analysis and comparison with animal data

Sup. Tab. 1: Primer sequences used in RT-qpCR.

Gene	Sequence
GUSB-fw	5'-TTA AAA GCA GCC CTG GTG AC-3'
GUSB-rv	5'-ATG TAG GTG GTG GGT GTC GT-3'
HPRT1- fw	5'-CCC CAC GAA GTG TTG GAT A-3'
HPRT1-rv	5'-AGC AGA TGG CCA CAG AAC T-3'
CYP2B6-fw	5'-TTC GGC GAT TCT CTG TGA CC-3'
CYP2B6-rv	5'-ATG AGG GCC CCC TTG GAT-3'
CYP3A4-fw	5'-TCA CAA ACC GGA GGC CTT TT-3'
CYP3A4-rv	5'-TGG TGA AGG TTG GAG ACA GC-3'
CYP8B1-fw	5'-GGG AGG TTC TTT GCA CTC AG-3'
CYP8B1-rv	5'-TAG TGG TGT GTC AGG GTC-3'
GAPDH-fw	5'-TTA AAA GCA GCC CTG GTG AC-3'
GAPDH-rv	5'-CTC TGC TCC TCC TGT TCG AC-'
ABCB1-fw	5'-GGG CTA GCA TGG ATC TTG AAG GGG ACC-3'
ABCB1-rv	5'-CCG GAT CCT CAC TGG CGC TTT GTT CC-3'
ABCC3-fw	5'-CCG CAT CCT GGT TTT AGA CG-3'
ABCC3-rv	5'-CAG GTA TCA AAC TGG GTG CG-3'
CASP3-fw	5'-ATC CAG TCG CTT TGT GCC AT-3'
CASP3-rv	5'-TCT GTT GCC ACC TTT CGG TTA-3'
CASP8-fw	5'-GAG GTT GAG GTG GGA GGA TT-3'
CASP8-rv	5'-ACG GGG TCT TGT TCT GTC AC-3'
GSTP1-fw	5'-CAT CTA CAC CAA CTA TGA GGC G-3'
GSTP1-rv	5'-AGC AGG GTC TCA AAA GGC TTC-3'
IL8-fw	5'GAC ATA CTC CAA ACC TTT CCA CC-3'
IL8-rv	5'-AAT TTC TGT GTT GGC GCA GTG-3'
PFKFB3-fw	5'-AAA AGT GTT CAA CGT CGG GG-3'
PFKFB3-rv	5'-CGA AAA CCG CAA TTT GTC CC-3'
SLC2A1-fw	5'-CCA GCA GCA AGA AGC TGA C-3'
SLC2A1-rv	5'-AGG ATG CTC TCC CCA TAG C-3'
UGT2B7-fw	5'-GCA ATG TTA TCA GGT TGA TCG GCA AAC AAT GGA ATC-3'
UGT2B7-rv	5'-GAC GTA TGG CTT ATT CGA AAC TCC TGG AAT TTT CAG-3'

Sup. Tab. 2: Comparison of $2^{-\Delta\Delta Ct}$ values obtained by RT-qPCR and PCR profiler array using cDNA generated from the same sample.

			HepaRG					
Gene			Cypro-conazole	Fluxa-pyroxad	Azoxy-strobin	Chloro-toluron	Thia-bendazole	2-Phenyl-phenol
CYP2B6	RT-qPCR	Mean	1.84	1.82	0.27	0.89	4.70	0.47
	Array	Mean	2.26	1.84	0.34	0.87	4.46	0.42
CYP3A4	RT-qPCR	Mean	1.88	1.67	0.38	0.46	1.17	0.47
	Array	Mean	2.31	1.95	0.37	0.50	1.36	0.43
CYP8B1	RT-qPCR	Mean	0.35	0.42	0.56	0.03	0.50	0.62
			RPTEC					
ABCB1	RT-qPCR	Mean	0.81	0.88	0.76	1.21	1.54	1.09
	Array	Mean	0.23	0.42	0.38	0.29	0.46	0.45

ABCC3	RT-qPCR	Mean	1.48	1.40	1.22	1.65	1.53	1.41
CASP3	RT-qPCR	Mean	3.21	2.92	1.62	3.40	5.05	0.81
CASP8	RT-qPCR	Mean	1.21	1.36	1.27	1.36	1.49	1.23
CYP1B1	RT-qPCR	Mean	1.45	0.12	0.30	0.63	1.50	0.41
GSTP1	RT-qPCR	Mean	0.91	1.02	1.16	1.13	0.95	1.16
	Array	Mean	0.72	0.82	1.00	0.71	0.80	0.92
IL8	RT-qPCR	Mean	1.37	1.95	0.76	2.71	2.51	0.70
PFKFB3	RT-qPCR	Mean	0.96	1.15	1.23	1.23	1.20	1.30
SLC2A1	RT-qPCR	Mean	1.80	2.39	2.22	2.62	1.00	1.54
UGT2B7	RT-qPCR	Mean	0.91	1.04	1.26	1.39	0.90	1.18

Sup. Tab. 3: All in vivo *effects identified by Nielsen et al. (2012) for the target organs liver and kidneys.*

Level 1: Toxicity to the organ	
**Liver**	**Kidneys**
Level 2: Phenomenological /specific effects on the organ	
Hepatocellular hypertrophy	Tubular cell degeneration / cell death
Hepatocellular fatty changes	Tubular fatty changes
Hepatocellular cell degeneration / cell death	Tubular hypertrophy / hyperplasia
Inflammation in the liver	Tubular neoplasms
Foci of cellular alteration in the liver	Tubular hyaline droplets
Neoplasms	Chronic progressive nephropathy (CPN)
Lesions of biliary epithelium	Alpha2u-globuline nephropathy
Porphyria	Glomerular cell degeneration / cell death
Cholestasis	Glomerular inflammation
Inclusions in hepatocytes	Inflammation
Karycytomegaly	Papillary cell degeneration / cell death
	Papillary hypertrophy / hyperplasia
	Pelvis hyperplasia
Level 3: Mode of Action	
Increase in phase I enzymes in the liver	Alpha2u-globulin
Oxidative stress	Calculi
Foci of cellular alteration and or liver neoplasms which may be caused by cytotoxicity	Crystals
Foci of cellular alteration and/or liver neoplasms which may be caused by hormonal changes	Oxidative stress
Level 4: Mechanism of Action	
Increase in CYP1A enzymes	Increased calcium in urine
Increase in CYP2A enzymes	
Increase in CYP2B enzymes	
Increase in CYP2E enzymes	
Increase in CYP3A enzymes	
Increase in CYP4A enzymes	
Degeneration and/or cell death which may be caused by porphyria	

Sup. Tab. 4: Combination of in vitro *effects derived from marker protein analysis in HepaRG cells with* in vivo *effects applying an evaluation matrix. N, no; Y, yes. +++, very strong: ≥ 2 conditions > 200 %; ++, strong: 1 condition > 200 %/ 2 conditions 150-200 %; +, medium: 1 condition 150-200 %.*

		Cyproconazole		Fluxapyroxad		Azoxystrobin		Chlorotoluron		Thiabendazole		2-Phenylphenol	
in vivo effect	*in vitro* effect	*in vivo*	*in vitro*	*in vivo*	*in vitro*	*in vivo*	*in vitro*	*in vivo*	*in vitro*	*in vivo*	*in vitro*	*in vivo*	*in vitro*
Foci of cellular alteration	Translation ↑	N		N		N		N	++	N		Y	
	Cell division ↑												
	Transcription ↑												
Hypertrophy	Translation ↑	Y		Y		Y		N	++	Y		N	
	Transcription ↑												
Neoplasms	Translation ↑	N		Y		N		N	++	N		Y	
	Cell division ↑												
	Transcription ↑												
Oxidative stress	Oxidative/ heat stress ↑						++					Y	
Hepatocellular cell degeneration/ death	Autophagy ↑	Y	+	Y		Y	++	N	+++	N	+++	N	
	Apoptosis ↑		++						+		++		

Sup. Tab. 5: Combination of in vitro *effects derived from marker protein analysis in RPTEC with* in vivo *effects applying an evaluation matrix. N, no; Y, yes. +++, very strong: ≥ 2 conditions > 200 %; ++, strong: 1 condition > 200 %/ 2 conditions 150-200 %; +, medium: 1 condition 150-200 %.*

		Cyproconazole		Fluxapyroxad		Azoxystrobin		Chlorotoluron		Thiabendazole		2-Phenylphenol	
in vivo effect	*in vitro* effect	*in vivo*	*in vitro*	*in vivo*	*in vitro*	*in vivo*	*in vitro*	*in vivo*	*in vitro*	*in vivo*	*in vitro*	*in vivo*	*in vitro*
Tubular neoplasms	Translation ↑	N	++	N	+++	N	+	Y	++	N		N	
	Cell division ↑												
	Transcription ↑												
Tubular hypertrophy/ hyperplasia	Translation ↑	N	++	N	+++	N	+	N	++	Y		Y	
	Cell division ↑												
	Transcription ↑												
Oxidative stress	Oxidative/ heat stress ↑											Y	
Tubular cell degeneration/ death	Autophagy ↑	N		N		N		N		Y		Y	
	Apoptosis ↑												

Sup. Tab. 6: Combination of in vitro *effects derived from marker protein analysis in HepaRG and RPTEC with* in vivo *effects applying an evaluation matrix. N, no; Y, yes. +++, very strong: ≥ 2 conditions > 200 %; ++, strong: 1 condition > 200 %/ 2 conditions 150-200 %; +, medium: 1 condition 150-200 %.*

		Cyproconazole			Fluxapyroxad			Azoxystrobin		
in vivo effect	*in vitro* effect	*in vivo*	HepaRG	RPTEC	*in vivo*	HepaRG	RPTEC	*in vivo*	HepaRG	RPTEC
Foci of cellular alteration	Translation ↑	N		++	N		+++	N		+
	Cell division ↑									
	Transcription ↑									
Hypertrophy	Translation ↑	Y		++	Y		+++	Y		+
	Transcription ↑									
Neoplasms	Translation ↑	N		++	Y		+++	N		+
	Cell division ↑									
	Transcription ↑									
Oxidative stress	Oxidative/ heat stress ↑								++	
Hepatocellular cell degeneration/ death	Autophagy ↑	Y	+		Y			Y	++	
	Apoptosis ↑		++							
Tubular neoplasms	Translation ↑	N		++	N		+++	N		+
	Cell division ↑									
	Transcription ↑									
Tubular hypertrophy/hyperplasia	Translation ↑	N		++	N		+++	N		+
	Cell division ↑									
	Transcription ↑									
Oxidative stress	Oxidative/ heat stress ↑								++	
Tubular cell degeneration/ death	Autophagy ↑	N	+		N			N	++	
	Apoptosis ↑		++							

		Chlorotoluron			Thiabendazole			2-Phenylphenol		
in vivo effect	*in vitro* effect	*in vivo*	HepaRG	RPTEC	*in vivo*	HepaRG	RPTEC	*in vivo*	HepaRG	RPTEC
Foci of cellular alteration	Translation ↑	N	++	++	N			Y		
	Cell division ↑									
	Transcription ↑									
Hypertrophy	Translation ↑	N	++	++	Y			N		

	Transcription ↑									
	Translation ↑		++	++						
Neoplasms	Cell division ↑	N			N			Y		
	Transcription ↑									
Oxidative stress	Oxidative/ heat stress ↑									
Hepatocellular cell degeneration/ death	Autophagy ↑	N	+++		N	+++		N		
	Apoptosis ↑		+			++				
	Translation ↑			++						
Tubular neoplasms	Cell division ↑	Y			N			N		
	Transcription ↑									
	Translation ↑			++						
Tubular hypertrophy/hyperplasia	Cell division ↑	N			Y			Y		
	Transcription ↑									
Oxidative stress	Oxidative/ heat stress ↑							Y		
Tubular cell degeneration/ death	Autophagy ↑	N	+++		Y	+++		Y		
	Apoptosis ↑		+			++				

Sup. Tab. 7: Combination of Ingenuity Pathway predictions from HepaRG dataset with in vivo *observations from the liver. N, no; Y, yes. +++, very strong: p-value ≤ 0.0005; ++, strong: p-value ≤ 0.005; +, medium: p-value ≤ 0.05.*

		Cyproconazole		Fluxapyroxad		Azoxystrobin		Chlorotoluron		Thiabendazole		2-Phenylphenol	
in vivo effect	Prediction	*in vivo*	*in vitro*	*in vivo*	*in vitro*	*in vivo*	*in vitro*	*in vivo*	*in vitro*	*in vivo*	*in vitro*	*in vivo*	*in vitro*
	Liver Proliferation								+++		+		
Neoplasms	Hepatocellular carcinoma, Liver Hyperplasia/ Hyperproliferation	N	+++	Y		N	+	N	+++	N	+	Y	
	Liver Hyperplasia/ Hyperproliferation		++				++		+++				
Cholestasis	Liver Cholestasis	Y	+++	N	+	N	++	N	+++	N		N	+

	Liver Cholestasis, Liver Inflammation/ Hepatitis		+						+				
Inflammation in the liver	Liver Cholestasis, Liver Inflammation/ Hepatitis	Y	+	N		Y		Y	+	N		N	
	Liver Inflammation/ Hepatitis		++						+++		++		+++
	Liver Inflammation/ Hepatitis, Liver Steatosis		++						+++		+		+
Hepatocellular cell degeneration/ death	Liver Necrosis/ Cell Death	Y	++	Y		Y		N	+	N		N	
Foci of cellular alteration in the liver	Liver Proliferation	N		N		N		N	+++	N	+	N	
Hepatocellular fatty changes	Liver Steatosis	Y	++	N		N		Y	+++	N	++	N	
	Liver Inflammation/ Hepatitis, Liver Steatosis		++						+++		+		+

Sup. Tab. 8: Combination of Ingenuity Pathway predictions from RPTEC dataset with in vivo *observations from the kidneys. N, no; Y, yes. +++, very strong: p-value ≤ 0.0005; ++, strong: p-value ≤ 0.005; +, medium: p-value ≤ 0.05.*

		Cyproconazole		Fluxapyroxad		Azoxystrobin		Chlorotoluron		Thiabendazole		2-Phenylphenol	
in vivo effect	Prediction	*in vivo*	*in vitro*	*in vivo*	*in vitro*	*in vivo*	*in vitro*	*in vivo*	*in vitro*	*in vivo*	*in vitro*	*in vivo*	*in vitro*
Glomerular cell degeneration	Glomerular Injury	N		N	+	N	++	N	++	N	+	N	
	Glomerular Injury, Renal Inflammation, Renal Nephritis		++		+		+		++		+		+
	Renal Necrosis/Cell Death		+++		++				++				+
Glomerular inflammation	Glomerular Injury, Renal Inflammation, Renal Nephritis	N	++	N	+	N	+	N	++	N	+	N	+

Inflammation	Glomerular Injury, Renal Inflammation, Renal Nephritis	N	++	N	+	N	+	N	++	Y	+	Y	+
	Renal Inflammation, Renal Nephritis												
Tubular cell degeneration/death	Renal Necrosis/Cell Death	N	+++	N	++	N		N	++	Y		Y	+
Tubular cell degeneration/ death	Renal Damage, Renal Tubule Injury	N		N		N		N		Y		Y	
Papillary cell degeneration/ death	Renal Necrosis/Cell Death	N	+++	N	++	N		N	++	N		Y	+
Tubular neoplasms	Renal Proliferation	N	+	N		N		Y	+	N		N	

Sup. Tab. 9: Combination of Ingenuity Pathway predictions from combined (HepaRG and RPTEC) dataset with in vivo *observations from the liver and the kidneys. N, no; Y, yes. +++, very strong: p-value ≤ 0.0005; ++, strong: p-value ≤ 0.005; +, medium: p-value ≤ 0.05.*

		Cyproconazole		Fluxapyroxad		Azoxystrobin		Chlorotoluron		Thiabendazole		2-Phenylphenol	
in vivo effect	Prediction	*in vivo*	*in vitro*	*in vivo*	*in vitro*	*in vivo*	*in vitro*	*in vivo*	*in vitro*	*in vivo*	*in vitro*	*in vivo*	*in vitro*
Cholestasis	Liver Cholestasis	Y	+++	N		N	++	N	+++	N	++	N	
	Liver Cholestasis,Liver Inflammation/ Hepatitis		+						+		+		
Inflammation in the liver	Liver Inflammation/ Hepatitis	Y	++	N		Y		Y	+++	N	++	N	+++
	Liver Inflammation/ Hepatitis, Liver Steatosis		+						+++				+
	Liver Cholestasis, Liver Inflammation/ Hepatitis		+						+		+		
Neoplasms	Liver Proliferation	N		Y		N		N	+++	N	++	Y	
	Liver Hyperplasia/ Hyperproliferation		+++		+		++		+++		+++		++
	Hepatocellular carcinoma, Liver		+++		+		++		+++		+++		++

	Hyperplasia/ Hyperproliferation												
Hepatocellular fatty changes	Liver Steatosis	Y	+++	N	++	N		Y	+++	N	+	N	+++
	Liver Inflammation/ Hepatitis,Liver Steatosis		+						+++				+
Hepatocellular cell degeneration/ death	Liver Necrosis/ Cell Death	Y	++	Y		Y		N	+	N		N	
Foci of cellular alteration in the liver	Liver Proliferation	N		N		N		N	+++	N	++	N	
Glomerular cell degeneration	Glomerular Injury	N		N	+	N	++	N	++	N		N	
	Glomerular Injury, Renal Inflammation, Renal Nephritis		+		+		+		+++		+		+
	Renal Necrosis/ Cell Death		++		++				+++				+
Glomerular inflammation	Glomerular Injury, Renal Inflammation, Renal Nephritis	N	+	N	+	N	+	N	+++	N	+	N	+
Inflammation	Renal Inflammation, Renal Nephritis	N		N		N		N	+++	Y		Y	++
	Glomerular Injury, Renal Inflammation, Renal Nephritis		+		+		+		+++		+		+
Tubular cell degeneration/ death	Renal Damage, Renal Tubule Injury	N		N		N		N	+	Y		Y	
	Renal Necrosis/ Cell Death		++		++				+++				+
Papillary cell degeneration/ death	Renal Necrosis/ Cell Death	N	++	N	++	N		N	+++	N		Y	+
Tubular neoplasms	Renal Proliferation	N		N		N		Y	+	N	+	N	+

Sup. Tab. 10: Combination of in vitro *effects derived from marker protein analysis in HepaRG and RPTEC and Ingenuity Pathway predictions from combined (HepaRG and RPTEC) dataset with* in vivo *observations from the liver and the kidneys. N, no; Y, yes. Protein: +++, very strong: ≥ 2 conditions > 200 %; ++, strong: 1 condition > 200 %/ 2 conditions 150-200 %; +, medium: 1 condition 150-200 %. mRNA: +++, very strong: p-value ≤ 0.0005; ++, strong: p-value ≤ 0.005; +, medium: p-value ≤ 0.05.*

			Cyproconazole				Fluxapyroxad				Azoxystrobin			
in vivo effect	Prediction	Cellular function	*in vivo*	mRNA	Protein		*in vivo*	mRNA	Protein		*in vivo*	mRNA	Protein	
					HepaRG	RPTEC			HepaRG	RPTEC			HepaRG	RPTEC
Neoplasms	Liver Hyperplasia/ Hyperproliferation	Translation ↑												
	Hepatocellular carcinoma, Liver Hyperplasia/ Hyperproliferation	Cell division ↑	N	+++		++	Y	+		+++	N	++		+
	Liver Proliferation	Transcription ↑		+++				+				++		
Foci of cellular alteration in the liver	Liver Proliferation	Translation ↑												
		Cell division ↑	N	+++		++	N	+		+++	N	++		+
		Transcription ↑												
Hepatocellular cell degeneration/ death	Liver Necrosis/ Cell Death	Autophagy ↑	Y	++	+		Y				Y		++	
		Apoptosis ↑			++									
Tubular cell degeneration/ death	Renal Damage, Renal Tubule Injury	Autophagy ↑	N		+		N				N		++	
	Renal Necrosis/ Cell Death	Apoptosis ↑		++	++			++						
Tubular neoplasms	Renal Proliferation	Transcription ↑												
		Translation ↑	N			++	N			+++	N			+
		Cell division ↑												

			Chlorotoluron				Thiabendazole				2-Phenylphenol			
in vivo effect	Prediction	Cellular function	*in vivo*	mRNA	Protein		*in vivo*	mRNA	Protein		*in vivo*	mRNA	Protein	
					HepaRG	RPTEC			HepaRG	RPTEC			HepaRG	RPTEC

Neoplasms	Liver Hyperplasia/ Hyperproliferation	Translation ↑		+++	++			++						
	Hepatocellular carcinoma, Liver Hyperplasia/ Hyperproliferation	Cell division ↑	N	+++		++	N	+++			Y	++		
	Liver Proliferation	Transcription ↑		+++				+++				++		
Foci of cellular alteration in the liver	Liver Proliferation	Translation ↑			++									
		Cell division ↑	N	+++		++	N	+++			N	++		
		Transcription ↑												
Hepatocellular cell degeneration/ death	Liver Necrosis/ Cell Death	Autophagy ↑			+++				+++					
		Apoptosis ↑	N	+	++		N		++		N			
Tubular cell degeneration/ death	Renal Damage, Renal Tubule Injury	Autophagy ↑	N	+	+++		Y		+++		Y			
	Renal Necrosis/ Cell Death	Apoptosis ↑		+++	++				++			+		
Tubular neoplasms	Renal Proliferation	Transcription ↑			++									
		Translation ↑	Y	+		++	N	+			N	+		
		Cell division ↑												

Equation 1

$$calculated\ organ\ dose\ (mg\ kg^{-1}) = \frac{dose\ organ\ (mg\ kg^{-1}\ organ)}{dosage\ (mg\ kg^{-1}\ bw)} \cdot LOAELorNOAEL(mg\ kg^{-1}\ bw)$$

Equation 2

$$calculated\ organ\ dose\ (\mu M) = \frac{calulcated\ organ\ dose\ (mg\ kg^{-1}\ organ) \cdot density(kg\ L^{-1})}{M\ (mg\ mmol^{-1})} \cdot 1000$$

Sup. Tab. 11: Comparison of calculated in vivo *organ concentrations with concentrations used in experiments with HepaRG and RPTEC.* In vivo *information from concordant sexes were used for calculation, where available, the LOAEL (otherwise NOAEL) from reliable short-term toxicity studies extracted from Draft Assessment Reports was used. All possible combinations of toxicokinetic studies and LOAEL/NOAEL studies were used to calculate the organ dose with equation 1. Organ density was set to 1 kg L^{-1} and used in equation 2 before mean and SD were calculated.*

Substance	Dosage (mg/kg bw)	Dose liver (mg/kg organ)	Dose kidney (mg/kg organ)	LOAEL/NOAEL (mg/kg bw)	Calc. dose liver (µM)	Calc. dose kidney (µM)	Used in HepaRG (µM)	Used in RPTEC (µM)
Cyproconazole	Single oral dose: 10	18.91	9.76	LAOEL<br>Rat (28): 25.3<br>Rat (90): 23.8 (m), 31.1 (f)	173 ± 20	89 ± 11	120<br>40	300<br>100
Fluxapyroxad	Single oral dose: 150	38.13 (m)<br>72.27 (f)	12.60 (m)<br>24.62 (f)	LOAEL<br>Mice (28 d):<br>112 (m), 150 (f)	363 ± 251	147 ±110	60<br>20	30<br>10
	Single oral dose: 7.5	11.93 (m),<br>13.74 (f)	4.98 (m),<br>5.80 (f)					
Azoxystrobin	Insufficient kinetic data			NOAEL<br>Rat (28 d): 53<br>Rat (90 d): 21	-	-	45<br>15	3<br>1
Chlorotoluron	Multiple intra-gastric doses 2.5	0.754	0.786	NOAEL<br>Mice (28 d):<br>>580 (m), >531 (f)	1419 ± 635	821 ± 36	750<br>250	900<br>300
	Single oral dose: 40	31.2	-					
Thiabendazole	Daily oral dose: 25	After 3 d:<br>8.24	After 3 d:<br>9.58	NOEL<br>Rat (28): <50<br>Rat (90): 10	49 ± 33	57 ± 38	300<br>100	900<br>300
2-phenylphenol	Single oral dose	1.05 ± 0.30 % dose/g	0.83 ± 0.35 % dose/g	LOAEL<br>Rat (90): 761	47 ± 11	37 ± 13	240<br>80	210<br>70
