# Supplementary material for: Comparative case study on NAMs: towards enhancing specific target organ toxicity analysis
Source: Arch Toxicol. 2024 Aug 29;98(11):3641–58. doi: 10.1007/s00204-024-03839-7 (PMC11489238; doi:10.1007/s00204-024-03839-7)
Supplement: Supplementary file 2 — Supplementary file2 (PDF 250 KB) [file 204_2024_3839_MOESM2_ESM.pdf]

Comparative Case Study on NAMs: Towards Enhancing Specific Target Organ Toxicity Analysis

Archives of Toxicology

Kristina Jochum[1], Andrea Miccoli[1,2,5], Cornelia Sommersdorf[3], Oliver Poetz[3,4], Albert Braeuning[5], Tewes Tralau[1], Philip Marx-Stoelting[1]

[1] German Federal Institute for Risk Assessment, Department of Pesticides Safety, Berlin, Germany

[2] National Research Council, Institute for Marine Biological Resources and Biotechnology (IRBIM), Ancona, Italy

[3] Signatope GmbH, Tübingen, Germany

[4] NMI Natural and Medical Sciences Institute at the University of Tübingen, Reutlingen, Germany

[5] German Federal Institute for Risk Assessment, Department of Food Safety, Berlin, Germany

philip.marx-stoelting@bfr.bund.de

Online Resource 2 Protocol for data analysis with GeneGlobe, IPA, GO and KEGG

GeneGlobe Webportal

A. Open web portal: https://geneglobe.qiagen.com/de
B. Create Qiagen Acocunt
C. Select **Analyze** (top left)
D. Start Analyzing your Data
    1. Select analysis type — PCR
    2. Select your analyte — mRNA/lncRNA
    3. Select your panel or assay — RT2 Profiler PCR Array 6 Assays
    4. **START YOUR ANALYSIS**
E. New Tab opens
    1. **Uplad/Select --> Uploaded Dataset**
        i. Product Type — Catalog Array
        ii. Species — Human
        iii. Catalog — select Array from dropdown
        iv. Plate Format — select from dropdown
        v. Upload File — download “sample Upload Data Excel Files”
           Durchsuchen --> select file
        vi. **Submit**
    2. **Setup --> Sample Manager**
        i. Check correct assignment
        ii. Set Cut-Off to 35
        iii. Select Compare each Test Group to Control Group ONLY
        iv. **Submit**
    3. **Setup --> Data QC**
        i. Check PCR Array Reproducibility and Reverse Transcription Efficiency Pass
        ii. Make sure gDNA contamination is close to 35 if it did not pass
    4. **Setup --> Normalization**
        i. Analysis name — name project
        ii. Normalization Method — from dropdown: Manual Selection
        iii. Normalized by Arithmetic Mean
        iv. Select all control genes
        v. Check that Arithmetic mean between groups does not vary too much
        vi. **Submit**
    5. **Export --> Export Excel Report**
        i. Select all options

        ii. **Submit**

F. Open File with Excel

    1. Copy Values from sheet Fold Regulation to upload file for IPA
    2. Copy values from sheet p-value to upload file for IPA
    3. Select header **data** in excel and transform the text to numbers with “Text in Spalten”

Ingenuity Pathway Analysis Software

A. Select **Create New** (top left) --> Core Analysis

B. Select **Upload** (top left) in window that opened up

C. Select upload file in appropriate format

    1. One column with ID
    2. Per condition: Fold change or regulation + p-value from GeneGlobe Export File https://geneglobe.qiagen.com/de/analyze

D. Dataset Uplaod

    1. Select File Format   Flexible Format
    2. Contains Column Headers   yes
    3. Select Identifier Type   first column as ID with dropdown menu
       Check assignment of Annotation (RefSeq)
    4. Array Platform used for experiments   not specified/applicable
    5. Use dropdown menu
        i. Assign observations to columns
        ii. Check if automatic selection of measurement is correct
        iii. Click Edit Observation Names to rename Observations with substance names
        iv. Select header Dataset Summary and check you do not have unmapped genes
        v. Select header Metadata to include information on incubation time, concentration, cell line, etc
    6. Select **save** (bottom right)

E. Save Dataset window

    1. Choose project or create new
    2. Enter name
    3. Add notes

F. Create Core Analysis window

    1. Select type of analysis   Expression Analysis
    2. Measurement type   Expr Fold Change
    3. **Next**

G. Create Gene Expression Analysis window
    1. General Settings nothing changed, direct and indirect relationships
    2. Networks nothing changed
    3. Node Types all
    4. Data sources All
    5. miRNA Confidence select all
    6. species human
    7. Tissue & Cell Line all
    8. Mutation all
    9. Set Cutoffs
        i. Expr Fold Change -1.5 Down & 1.5 Up
        ii. Expr p-value 0.05
        iii. Click **recalculate**
    10. Preview Dataset
        i. Check analysis ready molecules with drop down menu
        ii. Write down number of analysis ready molecules per observation
        iii. Check again you do not have unmapped molecules
    11. **Run Analysis** (bottom right)
H. Start Analysis window
    1. Project should be same as previously selected
    2. Analysis name should be same as previously entered
    3. Add notes
    4. **OK**
I. Wait till analysis is done
J. Project Manager window (left side)
    1. Select project
    2. Select observation --> double click
K. Expression Analysis window
    1. Select header Disease & Functions
    2. Select sub header Tox Functions
    3. From table on the bottom press icon to Export all
        i. Format .txt
        ii. Export Area all
        iii. **OK**

iv. **Save**

L. Continue for all observations

M. Open .txt with Excel

Gene Enrichment analysis with Gorilla

A. Run GO_skript.R to retrieve DEG and Background List

B. Open Gorilla: https://cbl-gorilla.cs.technion.ac.il/

1. Choose organism — Homo sapiens
2. Choose running mode: — Two unranked lists of genes
3. Paste a ranked list of gene/protein names
   i. Target set — DEG from GO_skript.R
   ii. Background set — generated with GO_skript.R
4. Choose an ontology: All
5. Click “Search Enriched GO terms”

C. Results page

1. Extract information on enriched GO Terms

KEGG Pathway analysis with ShinyGO 0.80

A. Run GO_skript.R to retrieve DEG and Background List

B. Open Shiny GO: http://bioinformatics.sdstate.edu/go/

1. Select a species — hsapiens_gene_enseml
2. Paste DEG per sub in open field
3. Click “Background (recommended)”: — Insert Background genes and DEG
4. Select Pathway database: — KEGG
5. FDR cutoff — 0.1
6. Pathways to show: — 100
7. Pathway size: Min — 2
8. Pathway size: Max — 2000

C. Extract Enrichment results to excel
