# Supplementary material for: Comparative case study on NAMs: towards enhancing specific target organ toxicity analysis
Source: Arch Toxicol. 2024 Aug 29;98(11):3641–58. doi: 10.1007/s00204-024-03839-7 (PMC11489238; doi:10.1007/s00204-024-03839-7)
Supplement: Supplementary file 4 — Supplementary file4 (PDF 4573 KB) [file 204_2024_3839_MOESM4_ESM.pdf]

Comparative Case Study on NAMs: Towards Enhancing Specific Target Organ Toxicity Analysis

Archives of Toxicology

Kristina Jochum[1], Andrea Miccoli[1,2,5], Cornelia Sommersdorf[3], Oliver Poetz[3,4], Albert Braeuning[5], Tewes Tralau[1], Philip Marx-Stoelting[1]

[1] German Federal Institute for Risk Assessment, Department of Pesticides Safety, Berlin, Germany

[2] National Research Council, Institute for Marine Biological Resources and Biotechnology (IRBIM), Ancona, Italy

[3] Signatope GmbH, Tübingen, Germany

[4] NMI Natural and Medical Sciences Institute at the University of Tübingen, Reutlingen, Germany

[5] German Federal Institute for Risk Assessment, Department of Food Safety, Berlin, Germany

philip.marx-stoelting@bfr.bund.de

Online Resource 4 Compilation of supplementary figures showing cytotoxicity, protein and mRNA results in more detail

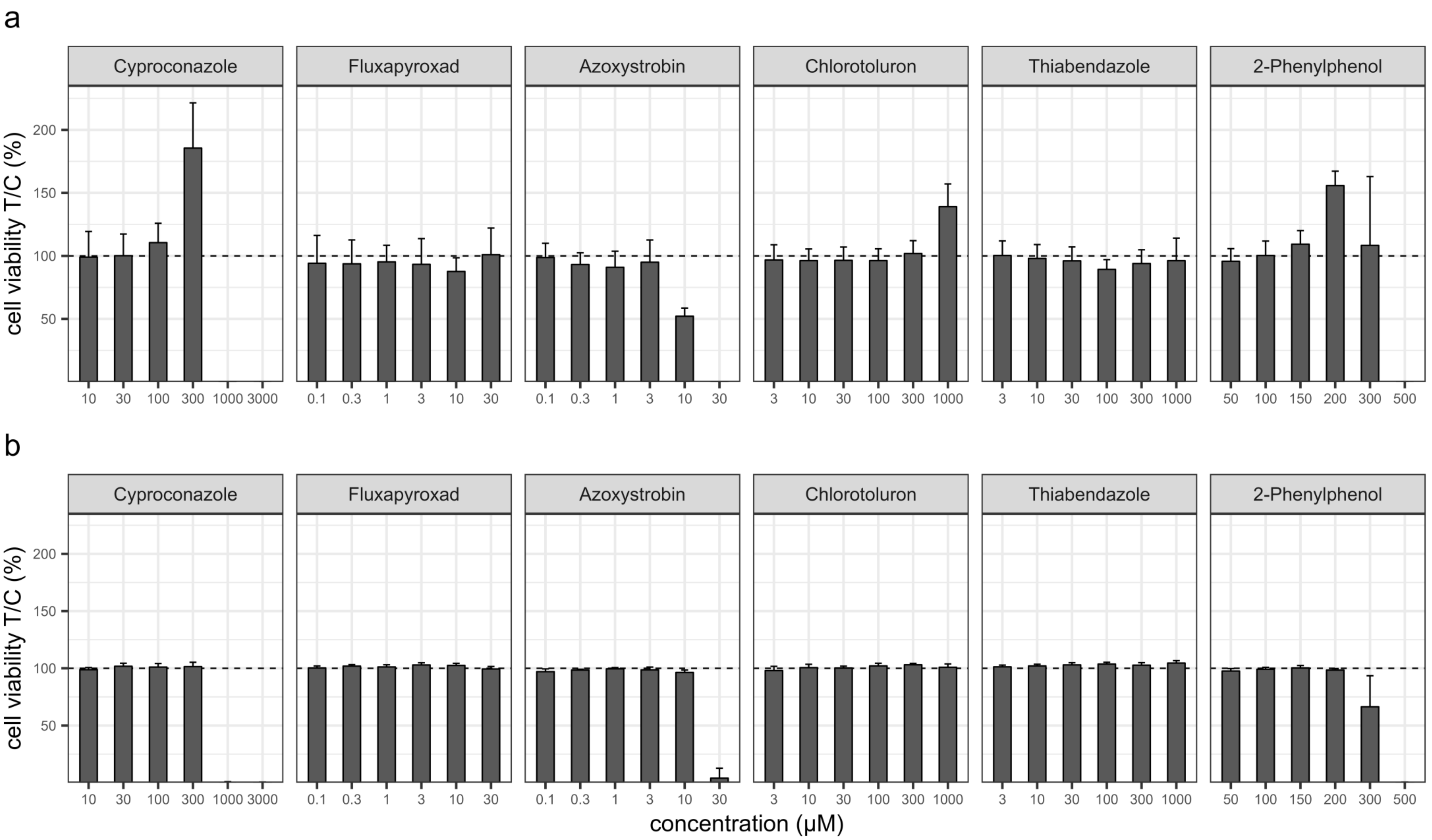

Sup. Fig 1 *Cell viability of RPTEC after 72 h incubation with the test substances at 6 different concentrations. Higher concentrations were limited by solubility of the substances. Panel a shows results from WST1 and panel b from neutral red uptake assay. At least 3 biological replicates were conducted in technical triplicates. Results are presented as means ± SD, normalized to the solvent controls (T/C) displayed by dashed lines*

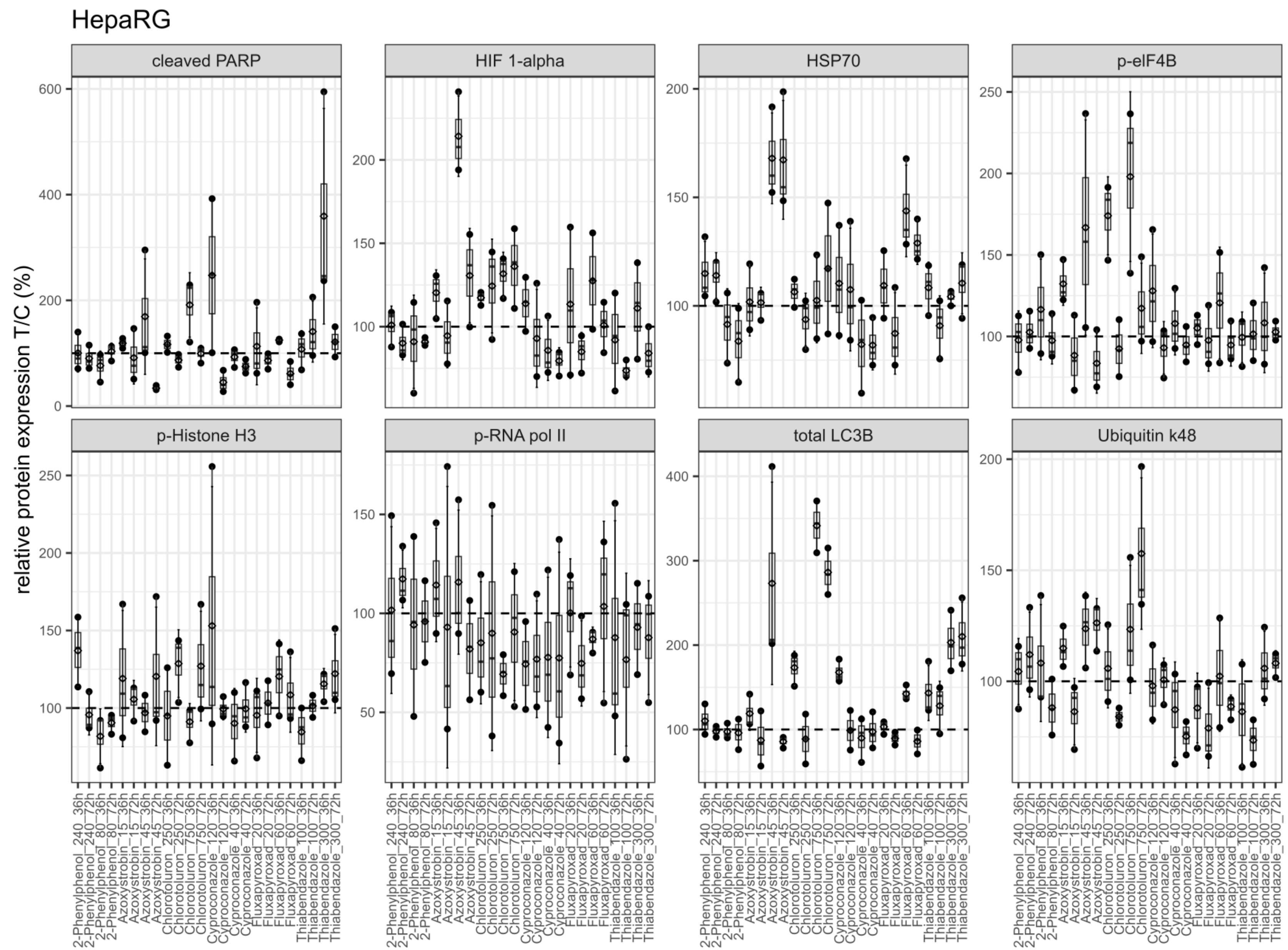

HepaRG
cleaved PARP
HIF 1-alpha
HSP70
p-eIF4B
p-Histone H3
p-RNA pol II
total LC3B
Ubiquitin k48
relative protein expression T/C (%)
2-Phenylphenol_240_36h
2-Phenylphenol_240_72h
2-Phenylphenol_80_36h
2-Phenylphenol_80_72h
Azoxystrobin_15_36h
Azoxystrobin_15_72h
Azoxystrobin_45_36h
Azoxystrobin_45_72h
Chlorotoluron_250_36h
Chlorotoluron_250_72h
Chlorotoluron_750_36h
Chlorotoluron_750_72h
Cyproconazole_120_36h
Cyproconazole_120_72h
Cyproconazole_40_36h
Cyproconazole_40_72h
Fluxapyroxad_20_36h
Fluxapyroxad_20_72h
Fluxapyroxad_60_36h
Fluxapyroxad_60_72h
Thiabendazole_100_36h
Thiabendazole_100_72h
Thiabendazole_300_36h
Thiabendazole_300_72h

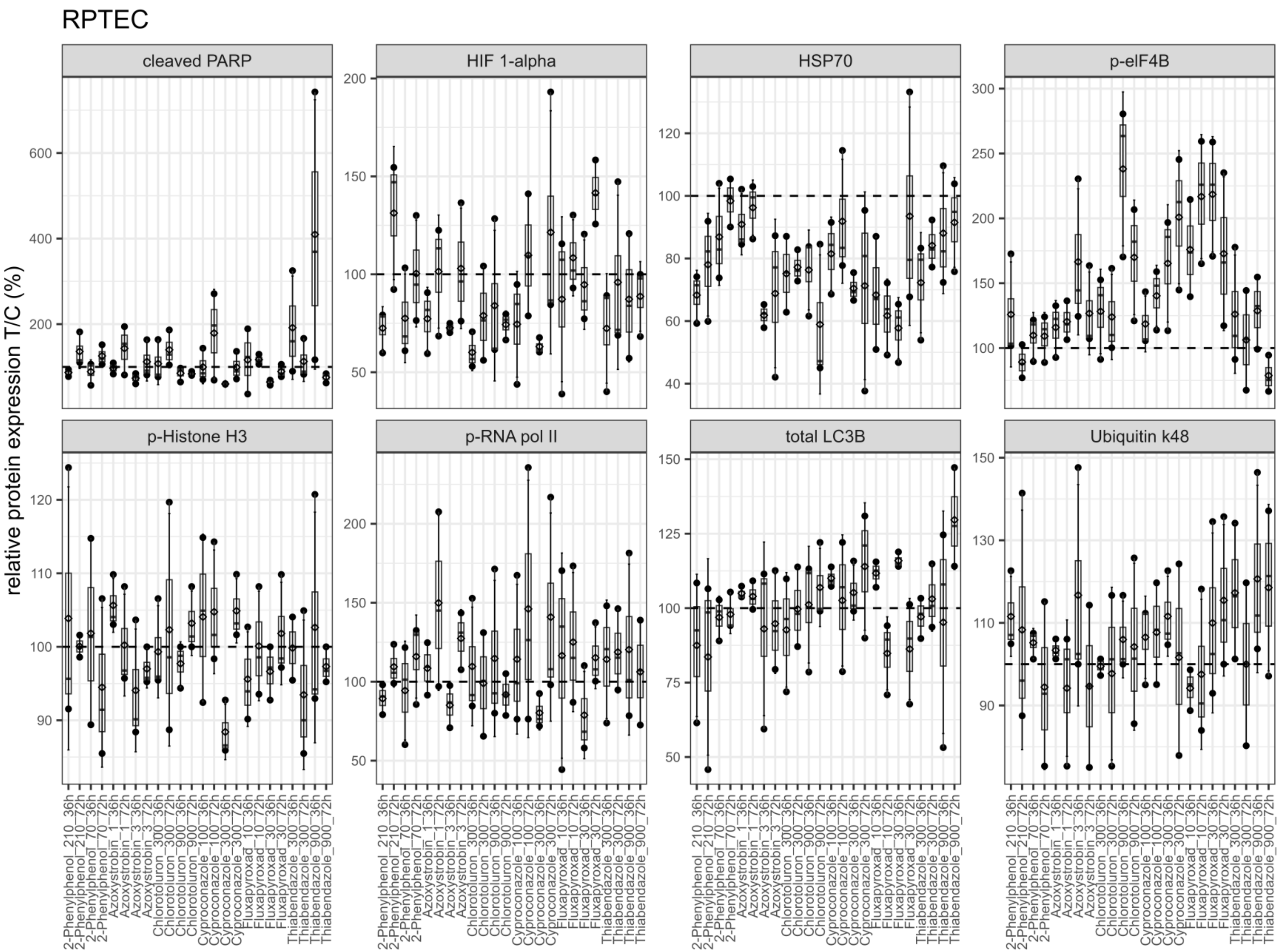

***Sup. Fig 2** Effects on protein expression of selected markers observed in HepaRG cells (top) and RPTEC (bottom) after 36 h and 72 h of incubation with the test substances using a multiplexed microsphere-based sandwich immunoassay panel. Points represent individual data points of three biological replicates related to solvent control (T/C). Diamonds represent mean value and standard deviation is indicated by error bar*

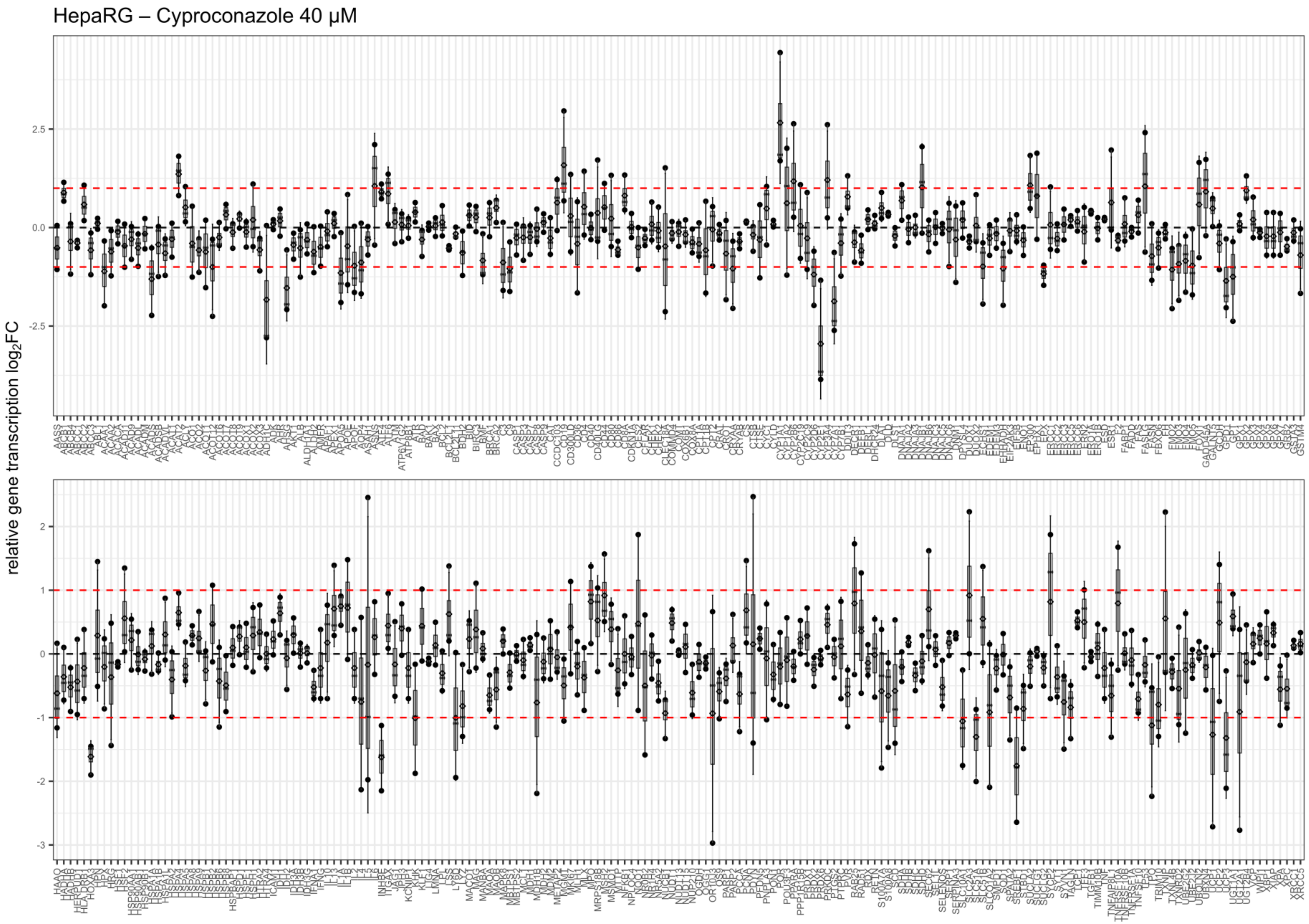
HepaRG – Cyproconazole 40 µM
relative gene transcription log2FC
2.5
0.0
-2.5
2
1
0
-1
-2
-3

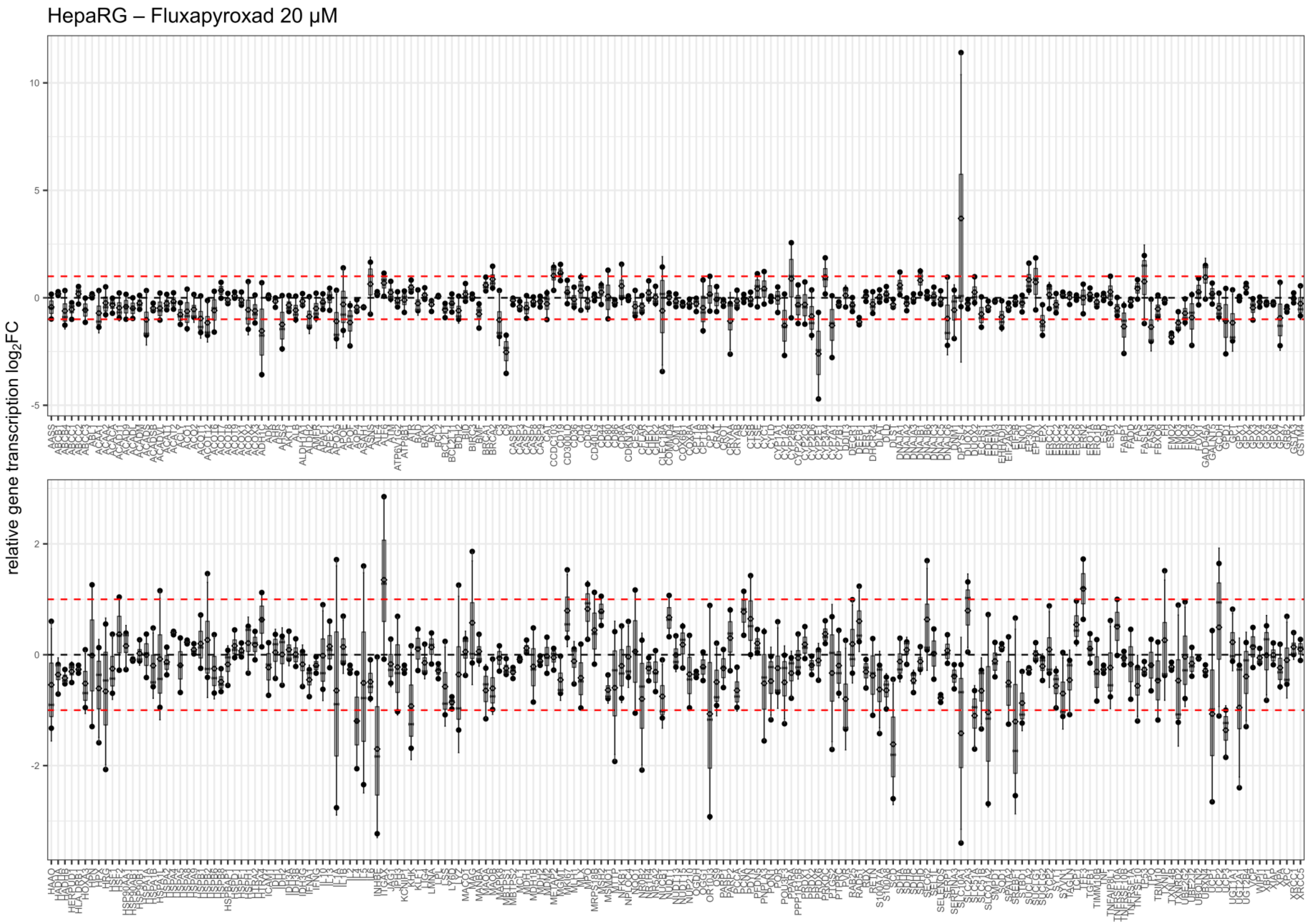
HepaRG – Fluxapyroxad 20 µM
relative gene transcription log2FC
10
5
0
-5
2
0
-2

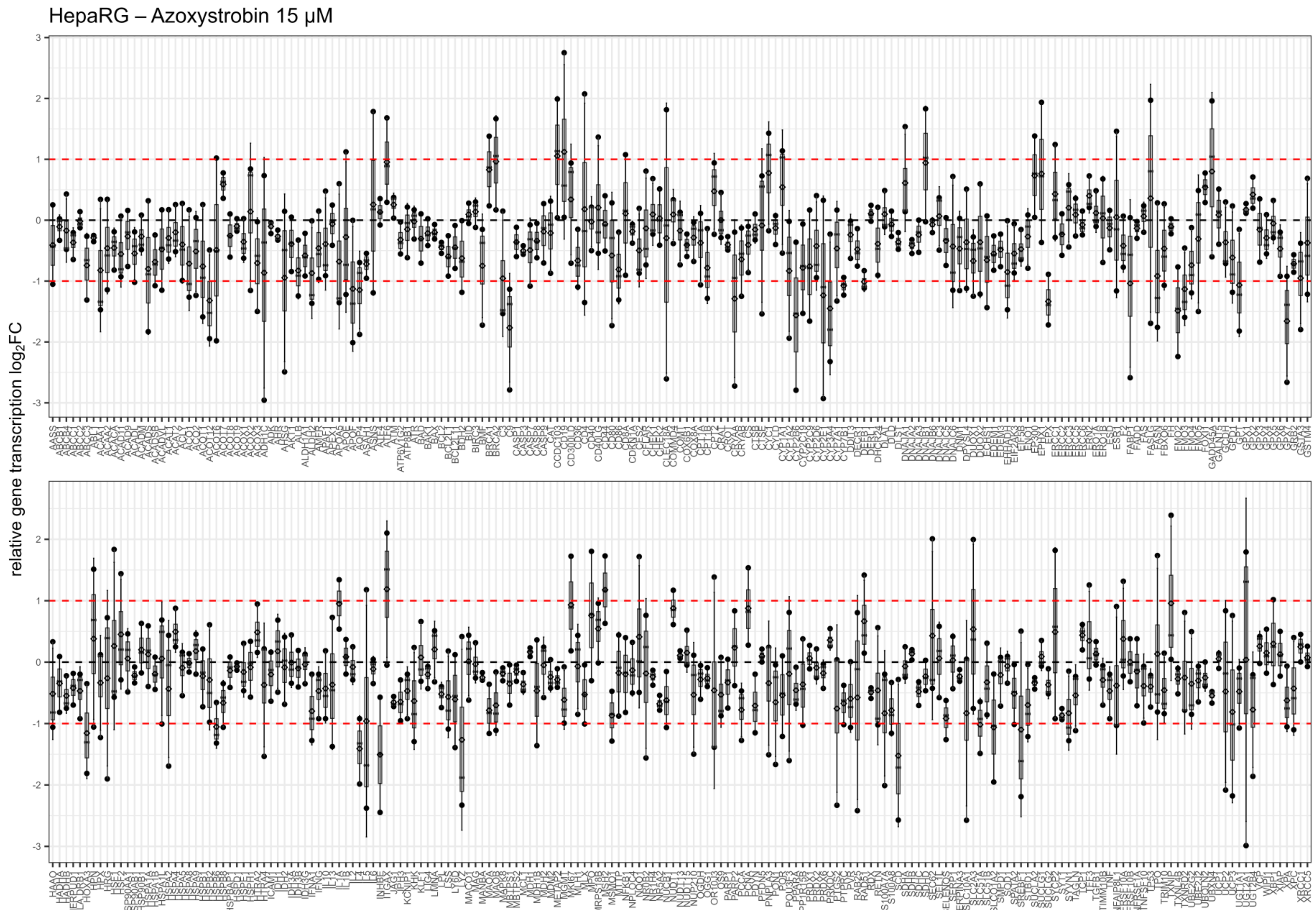
HepaRG – Azoxystrobin 15 µM
relative gene transcription log2FC

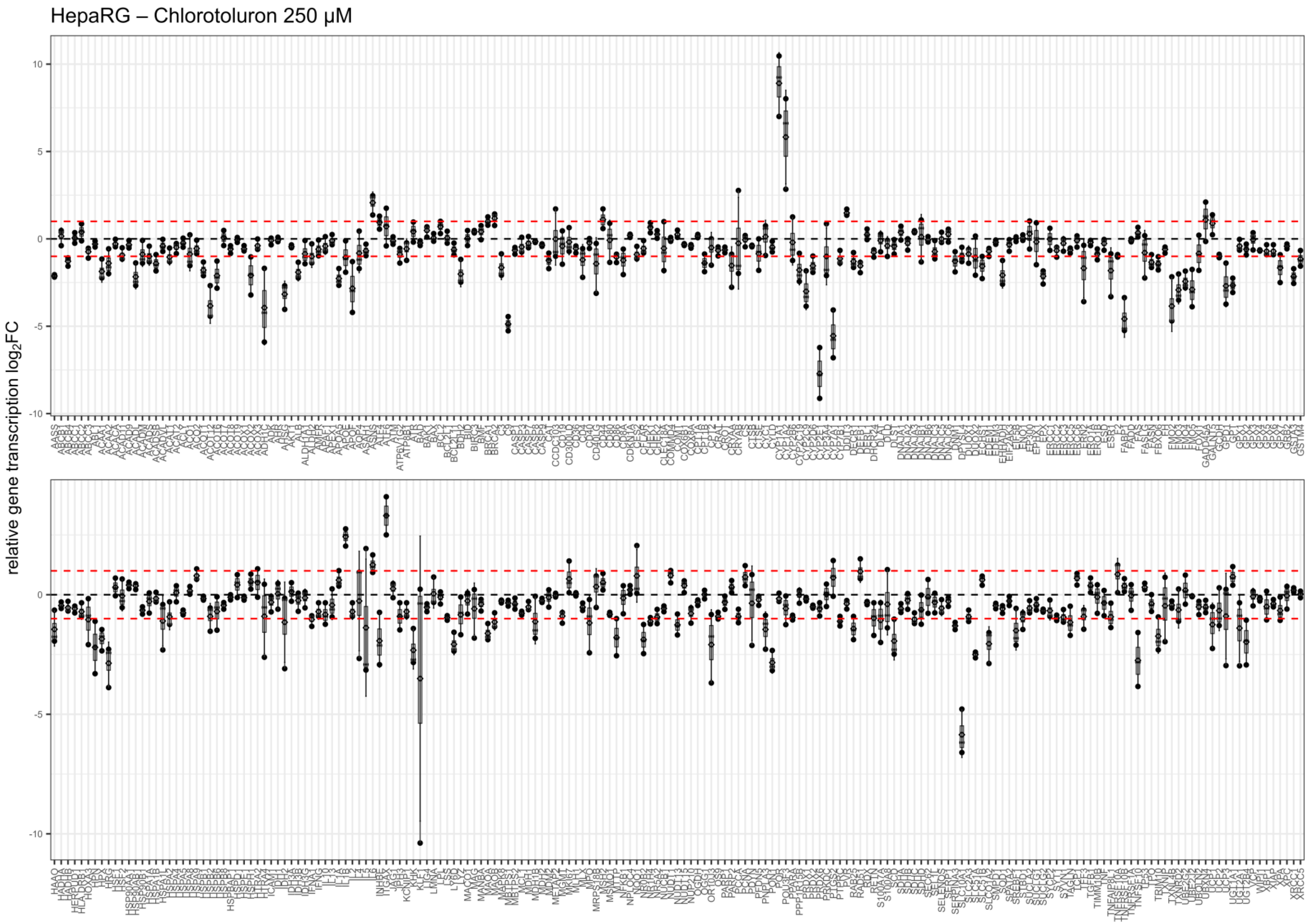
HepaRG – Chlorotoluron 250 µM
relative gene transcription log2FC
10
5
0
-5
-10

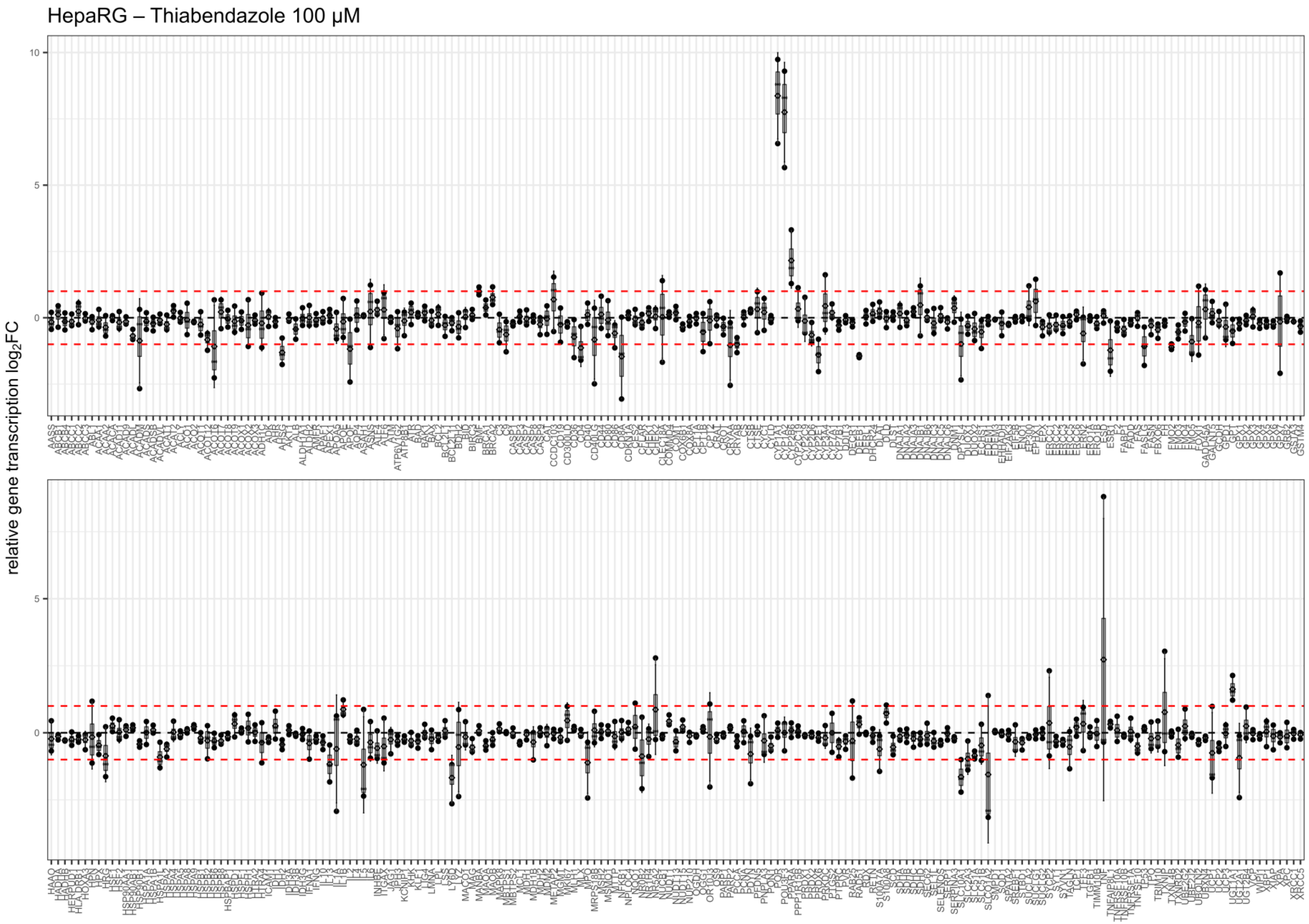
HepaRG – Thiabendazole 100 µM
relative gene transcription log_2FC
10
5
0
5
0

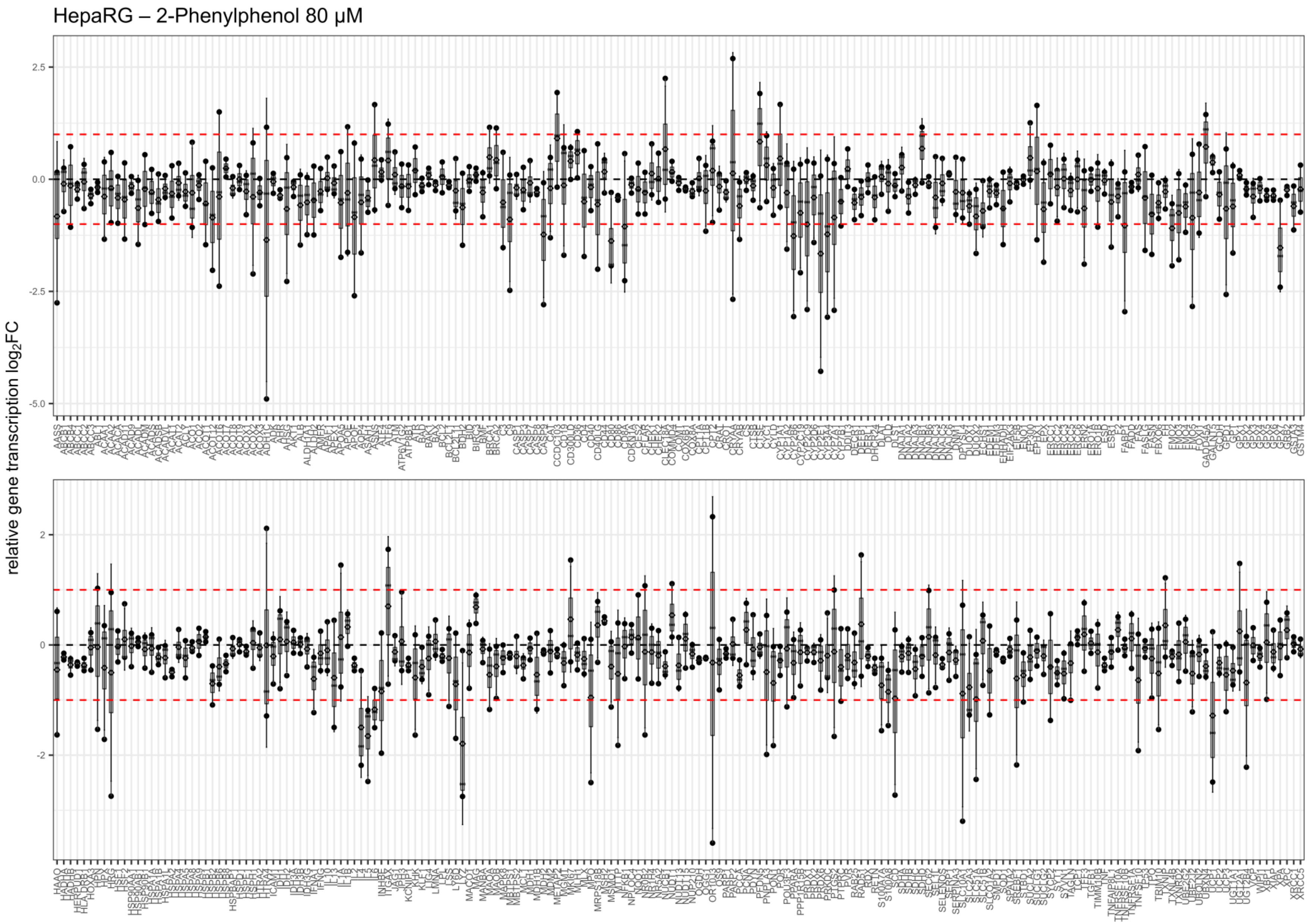

Sup. Fig 3 *Relative quantities of mRNA transcript levels observed after 36 h exposure of HepaRG cells to non-cytotoxic concentrations of the test substances using the Human Molecular Toxicology PathwayFinder RT2 ProfilerTM PCR Array. Data evaluation was performed using 2-ΔΔCt method, according to Livak and Schmittgen (2001). All target genes were normalized to 5 housekeeping genes. Points represent individual data points of three biological replicates as -ΔΔCT values. Diamonds represent mean value and standard deviation is indicated by error bars*

RPTEC

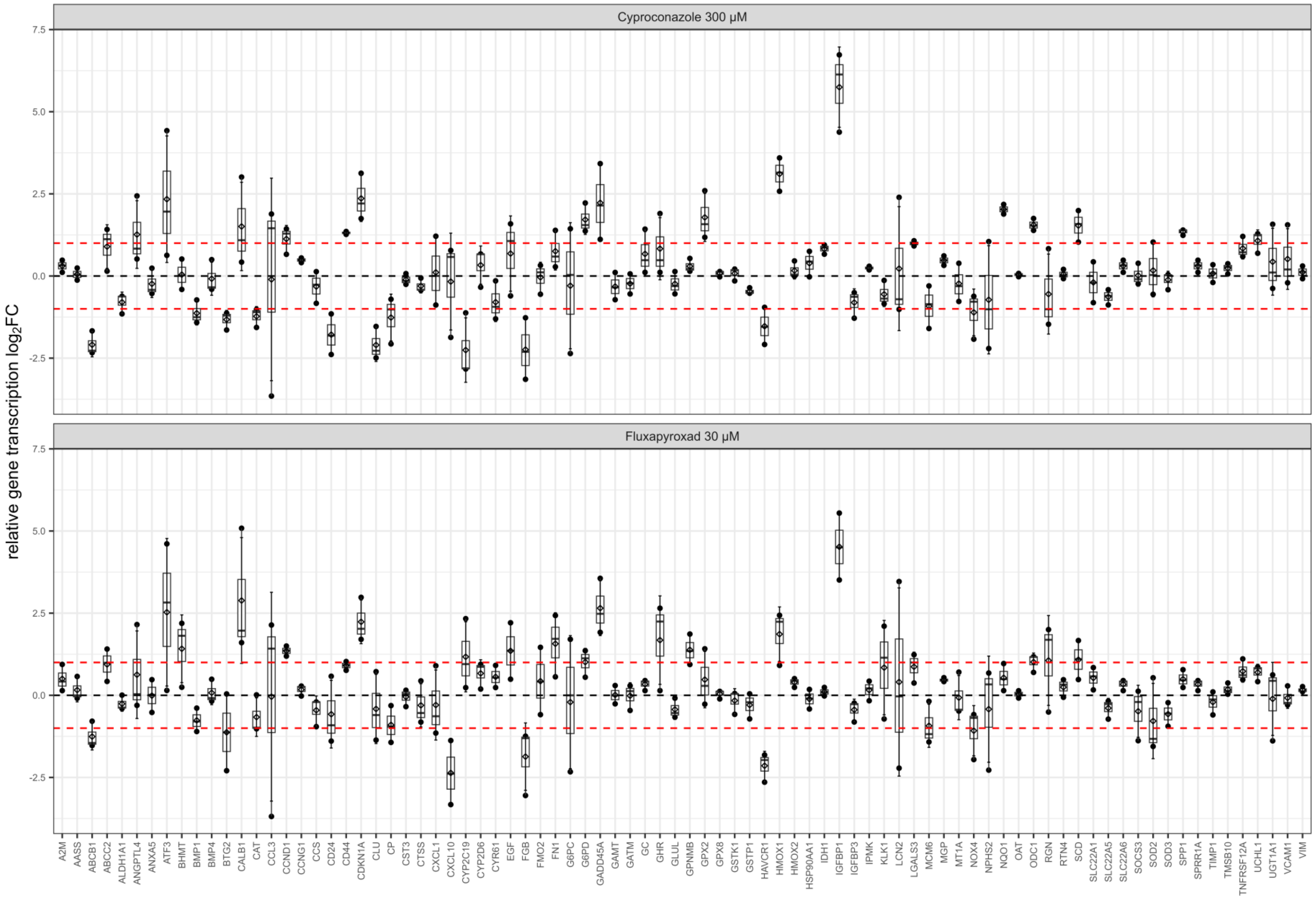
Cyproconazole 300 µM
Fluxapyroxad 30 µM
relative gene transcription log2FC
7.5
5.0
2.5
0.0
-2.5
A2M AASS ABCB1 ABCC2 ALDH1A1 ANGPTL4 ANXA5 ATF3 BHMT BMP1 BMP4 BTG2 CALB1 CAT CCL3 CCND1 CCNG1 CCS CD24 CD44 CDKN1A CLU CP CST3 CTSS CXCL1 CXCL10 CYP2C19 CYP2D6 CYR61 EGF FGB FMO2 FN1 G6PC G6PD GADD45A GAMT GATM GC GHR GLUL GPNMB GPX2 GPX8 GSTK1 GSTP1 HAVCR1 HMOX1 HMOX2 HSP90AA1 IDH1 IGFBP1 IGFBP3 IPMK KLK1 LCN2 LGALS3 MCM6 MGP MT1A NOX4 NPHS2 NQO1 OAT ODC1 RGN RTN4 SCD SLC22A1 SLC22A5 SLC22A6 SOCS3 SOD2 SOD3 SPP1 SPRR1A TIMP1 TMSB10 TNFRSF12A UCHL1 UGT1A1 VCAM1 VIM

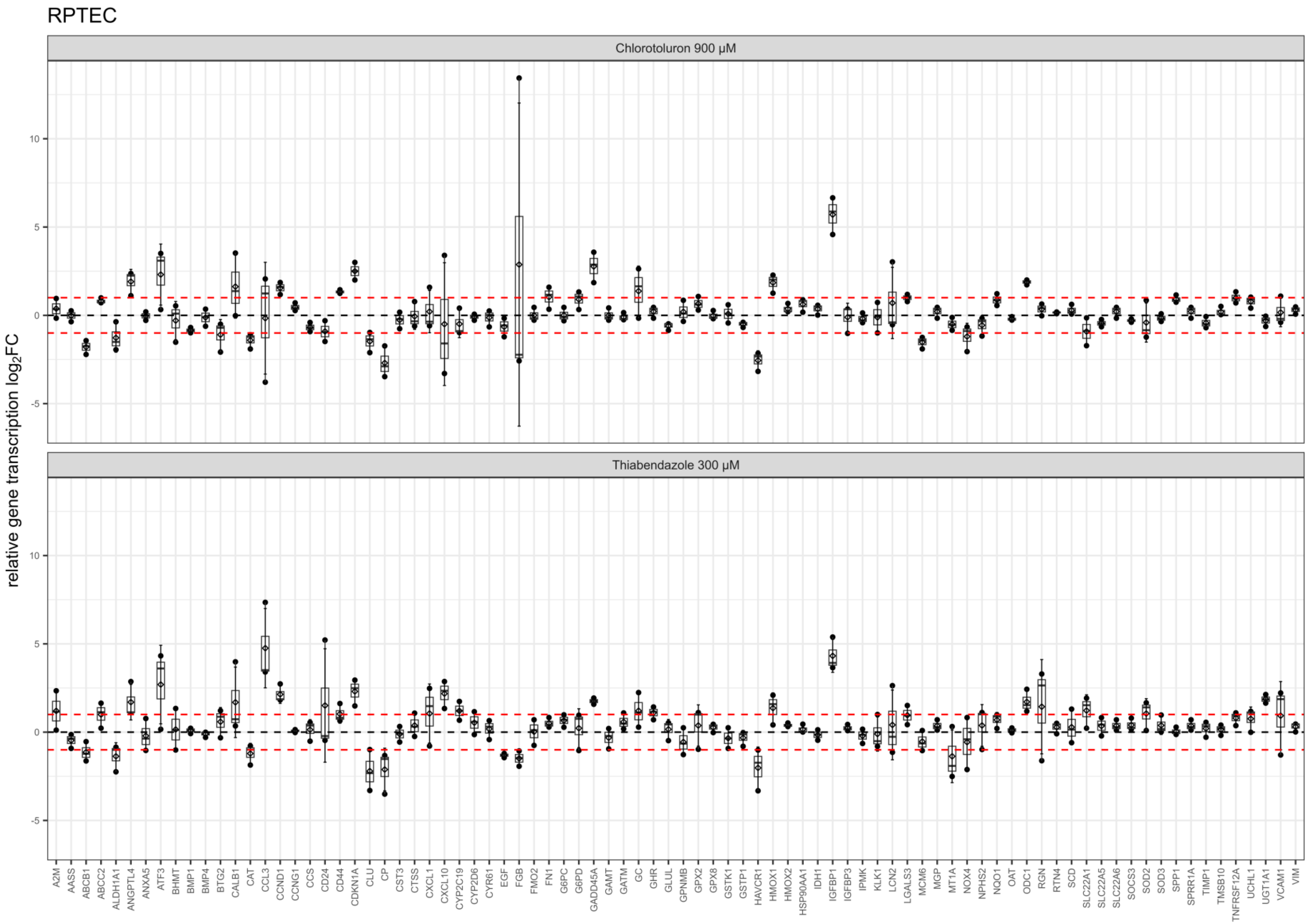

RPTEC
Chlorotoluron 900 µM
Thiabendazole 300 µM
relative gene transcription log_2FC
10
5
0
-5
A2M AASS ABCB1 ABCC2 ALDH1A1 ANGPTL4 ANXA5 ATF3 BHMT BMP1 BMP4 BTG2 CALB1 CAT CCL3 CCND1 CCNG1 CCS CD24 CD44 CDKN1A CLU CP CST3 CTSS CXCL1 CXCL10 CYP2C19 CYP2D6 CYR61 EGF FGB FMO2 FN1 G6PC G6PD GADD45A GAMT GATM GC GHR GLUL GPNMB GPX2 GPX8 GSTK1 GSTP1 HAVCR1 HMOX1 HMOX2 HSP90AA1 IDH1 IGFBP1 IGFBP3 IPMK KLK1 LCN2 LGALS3 MCM6 MGP MT1A NOX4 NPHS2 NQO1 OAT ODC1 RGN RTN4 SCD SLC22A1 SLC22A5 SLC22A6 SOCS3 SOD2 SOD3 SPP1 SPRR1A TIMP1 TMSB10 TNFRSF12A UCHL1 UGT1A1 VCAM1 VIM

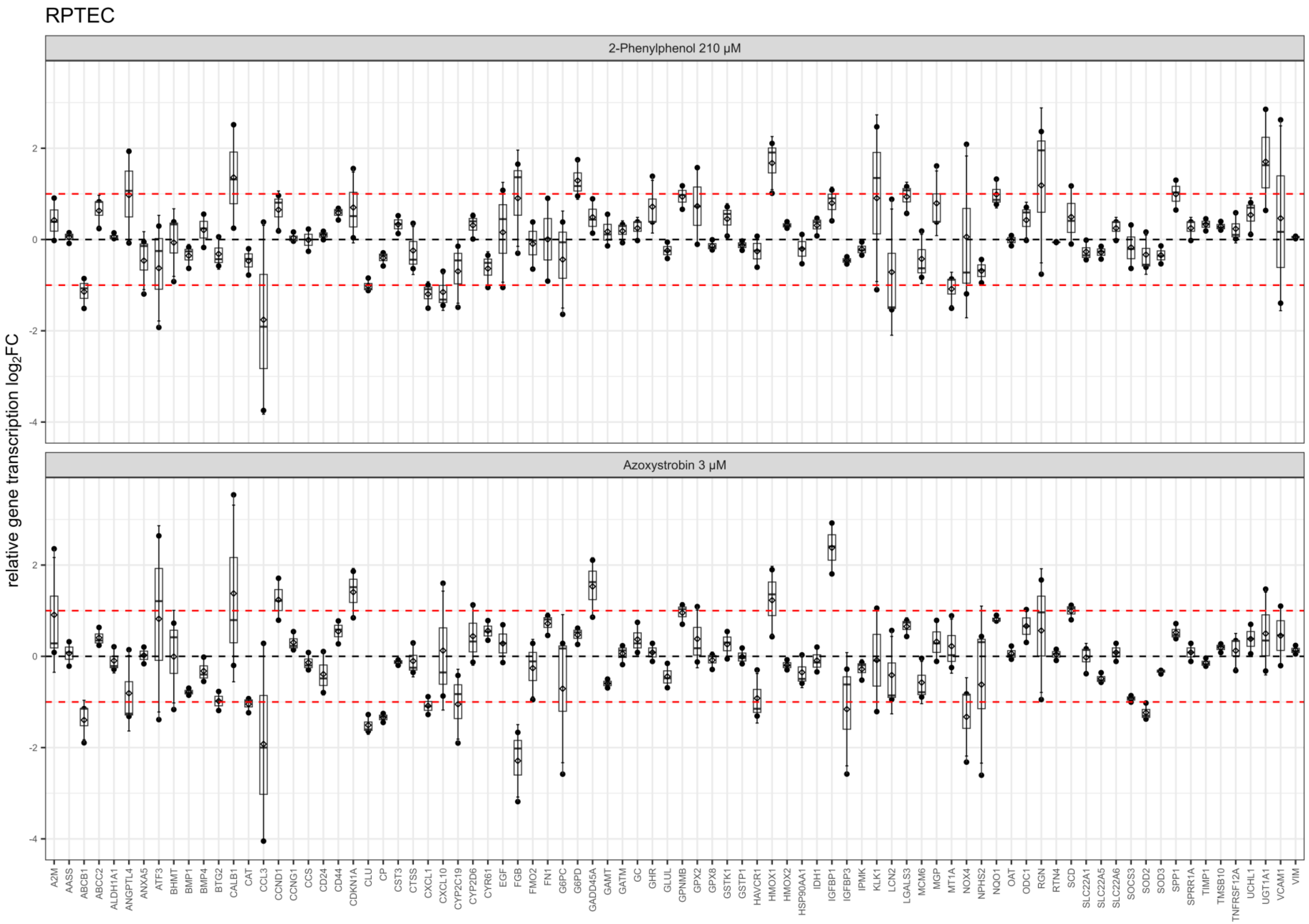

Sup. Fig 4 *Relative quantities of mRNA transcript levels observed after 36 h exposure of HepaRG cells to non-cytotoxic concentrations of the test substances using the Human Molecular Toxicology PathwayFinder RT2 ProfilerTM PCR Array. Data evaluation was performed using 2-ΔΔCt method, according to Livak and Schmittgen (2001). All target genes were normalized to 5 housekeeping genes. Points represent individual data points of three biological replicates as -ΔΔCT values. Diamonds represent mean value and standard deviation is indicated by error bars*
